# Supplementary figures and images for: The Filament Sensor for Near Real-Time Detection of Cytoskeletal Fiber Structures
Source: PLoS One. 2015 May 21;10(5):e0126346. doi: 10.1371/journal.pone.0126346 (PMC4440737; doi:10.1371/journal.pone.0126346)

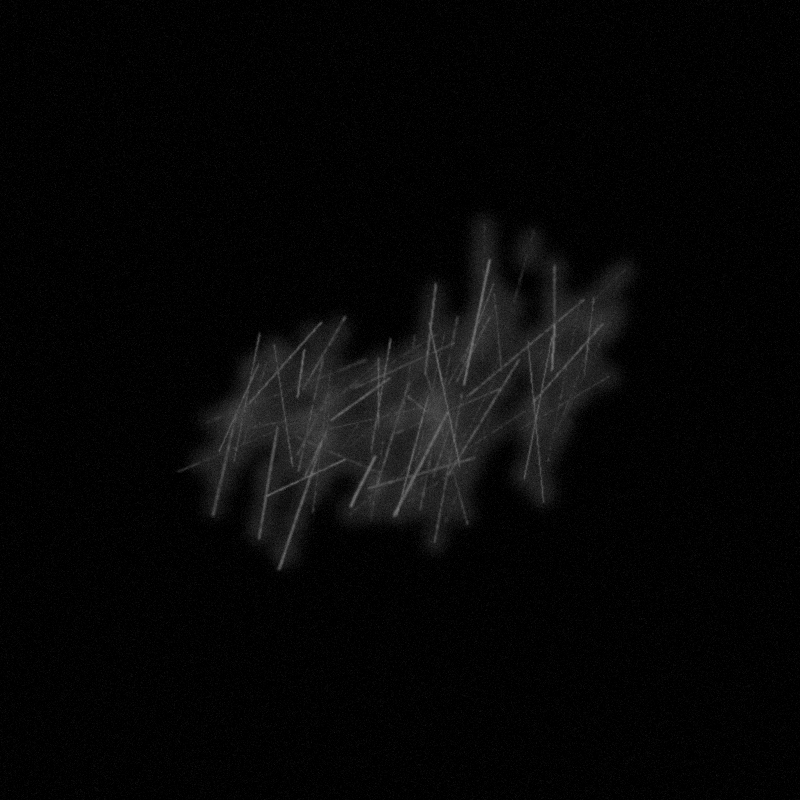

Supplement: S1 File — This comprises the FilamentSensor, the benchmark database, the output of the compared programs, several auxiliary scripts, and the evaluation results of the program outputs. (ZIP) [file pone.0126346.s001.zip › supporting_information/ground_truth/simulated/simulated_cell_02.png]

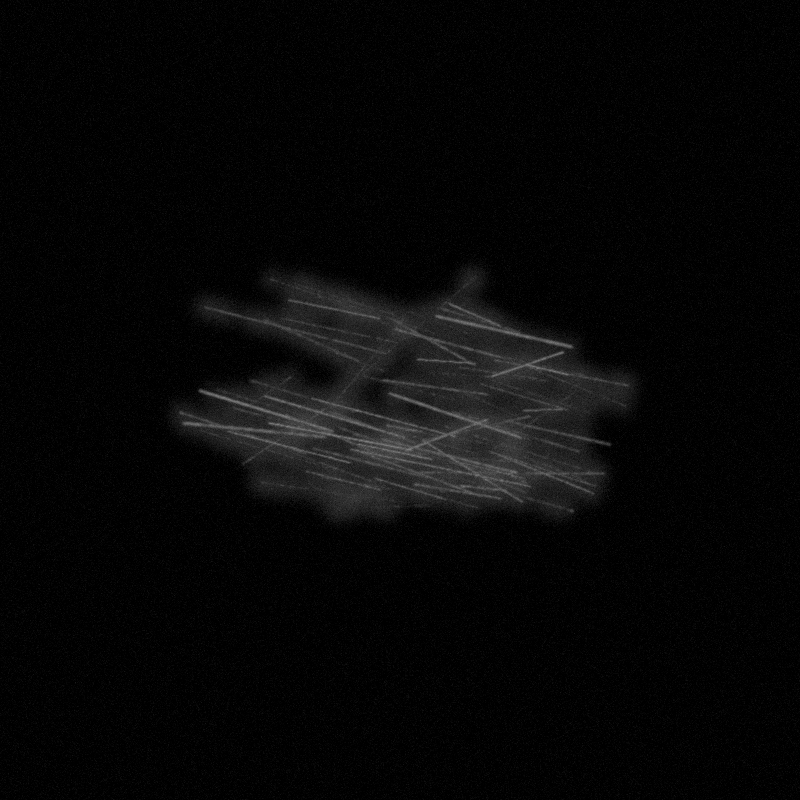

Supplement: S1 File — This comprises the FilamentSensor, the benchmark database, the output of the compared programs, several auxiliary scripts, and the evaluation results of the program outputs. (ZIP) [file pone.0126346.s001.zip › supporting_information/ground_truth/simulated/simulated_cell_01.png]

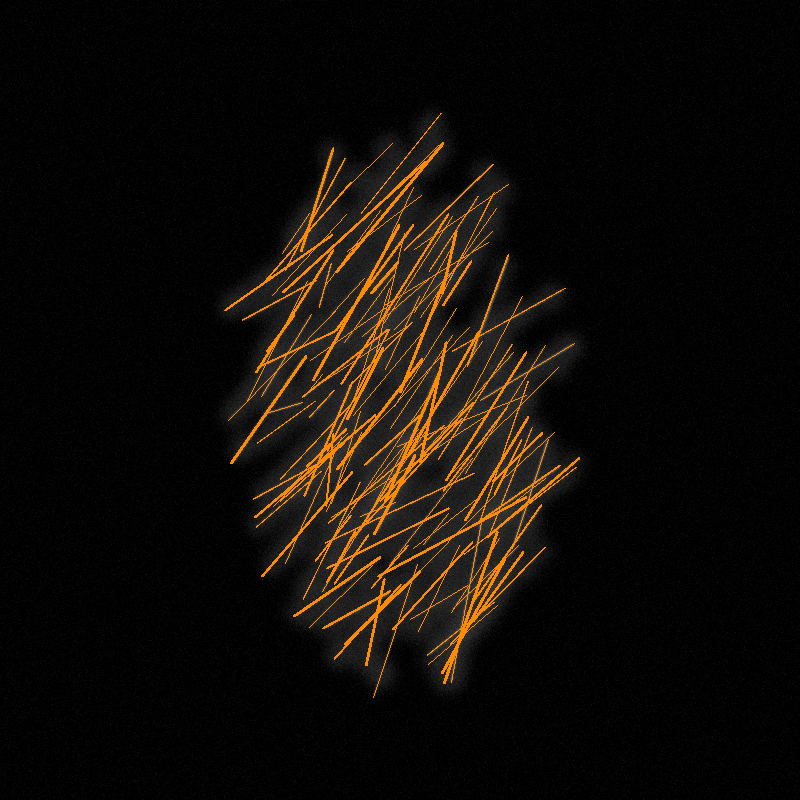

Supplement: S1 File — This comprises the FilamentSensor, the benchmark database, the output of the compared programs, several auxiliary scripts, and the evaluation results of the program outputs. (ZIP) [file pone.0126346.s001.zip › supporting_information/ground_truth/simulated/ground_truth_simulated_img/simulated_cell_07_png_filaments.png]

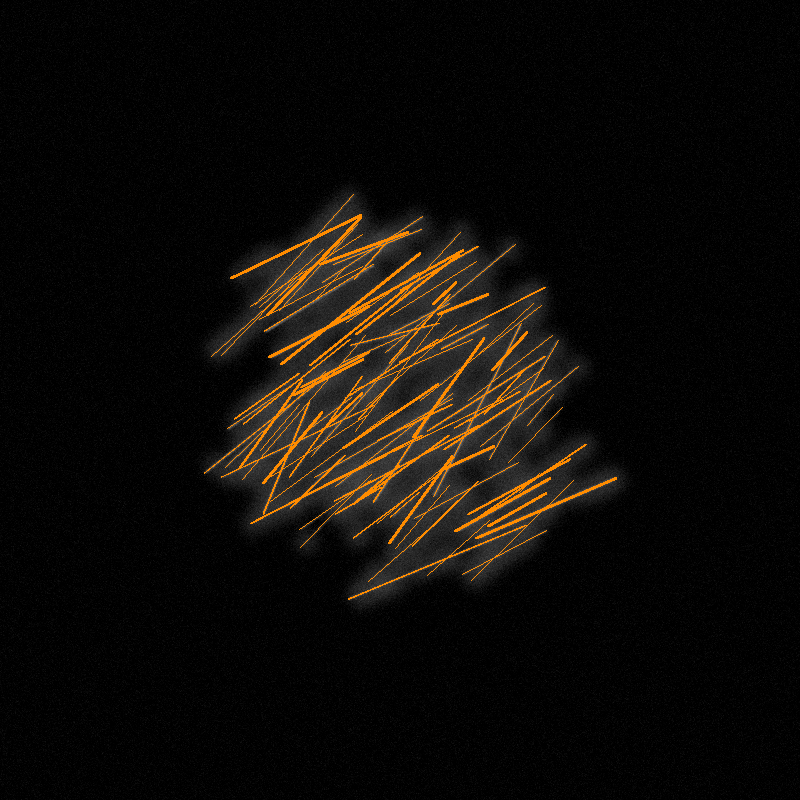

Supplement: S1 File — This comprises the FilamentSensor, the benchmark database, the output of the compared programs, several auxiliary scripts, and the evaluation results of the program outputs. (ZIP) [file pone.0126346.s001.zip › supporting_information/ground_truth/simulated/ground_truth_simulated_img/simulated_cell_05_png_filaments.png]

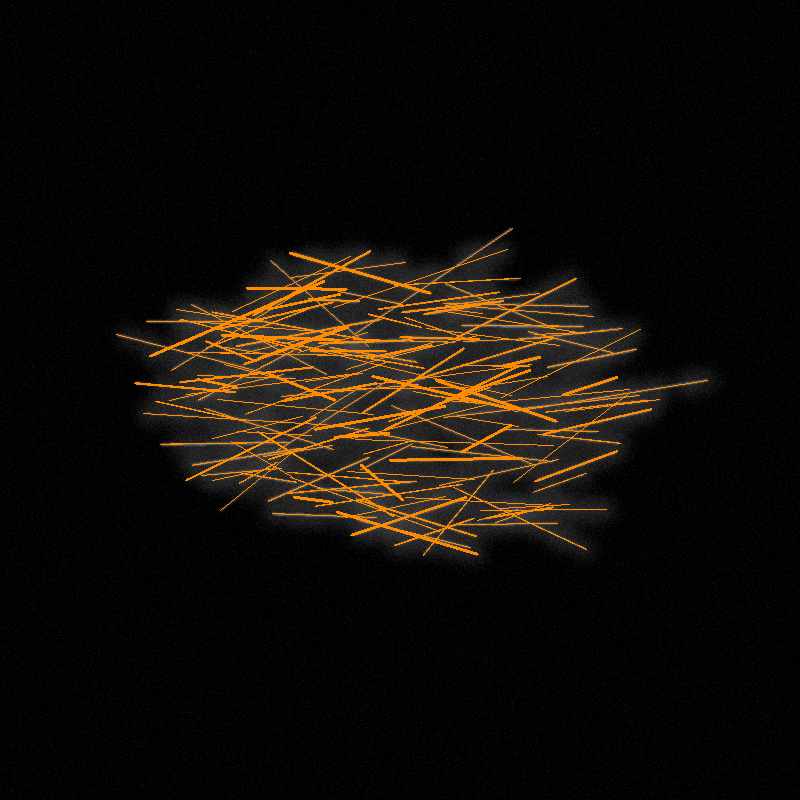

Supplement: S1 File — This comprises the FilamentSensor, the benchmark database, the output of the compared programs, several auxiliary scripts, and the evaluation results of the program outputs. (ZIP) [file pone.0126346.s001.zip › supporting_information/ground_truth/simulated/ground_truth_simulated_img/simulated_cell_09_png_filaments.png]

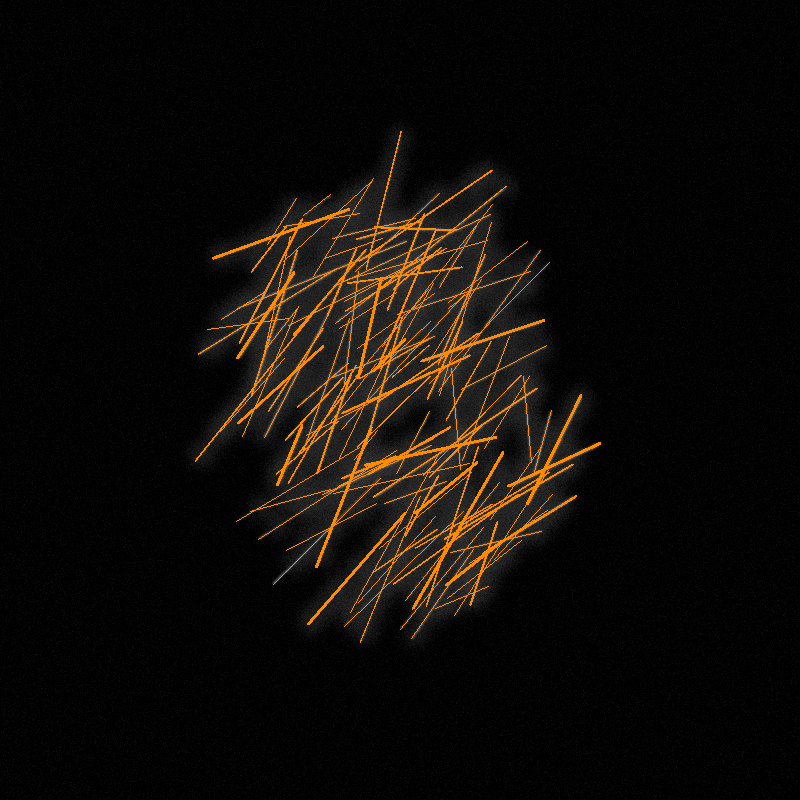

Supplement: S1 File — This comprises the FilamentSensor, the benchmark database, the output of the compared programs, several auxiliary scripts, and the evaluation results of the program outputs. (ZIP) [file pone.0126346.s001.zip › supporting_information/ground_truth/simulated/ground_truth_simulated_img/simulated_cell_06_png_filaments.png]

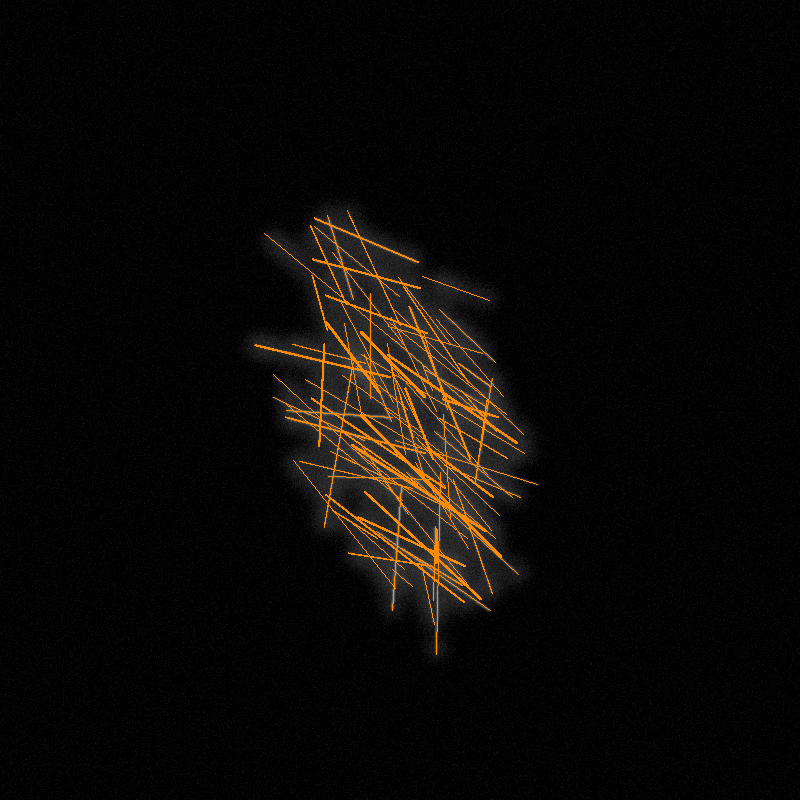

Supplement: S1 File — This comprises the FilamentSensor, the benchmark database, the output of the compared programs, several auxiliary scripts, and the evaluation results of the program outputs. (ZIP) [file pone.0126346.s001.zip › supporting_information/ground_truth/simulated/ground_truth_simulated_img/simulated_cell_04_png_filaments.png]

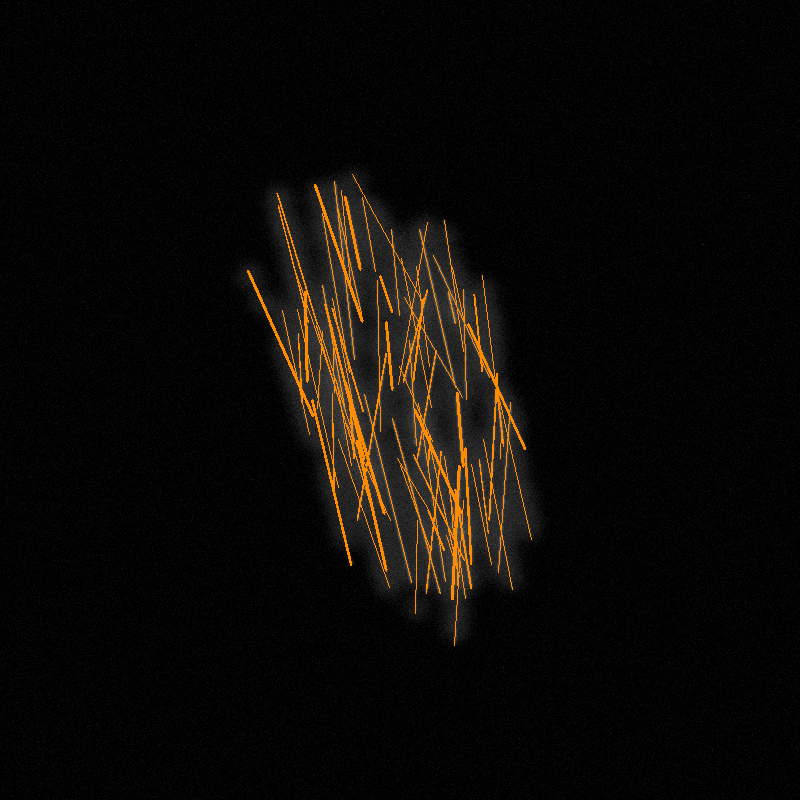

Supplement: S1 File — This comprises the FilamentSensor, the benchmark database, the output of the compared programs, several auxiliary scripts, and the evaluation results of the program outputs. (ZIP) [file pone.0126346.s001.zip › supporting_information/ground_truth/simulated/ground_truth_simulated_img/simulated_cell_08_png_filaments.png]

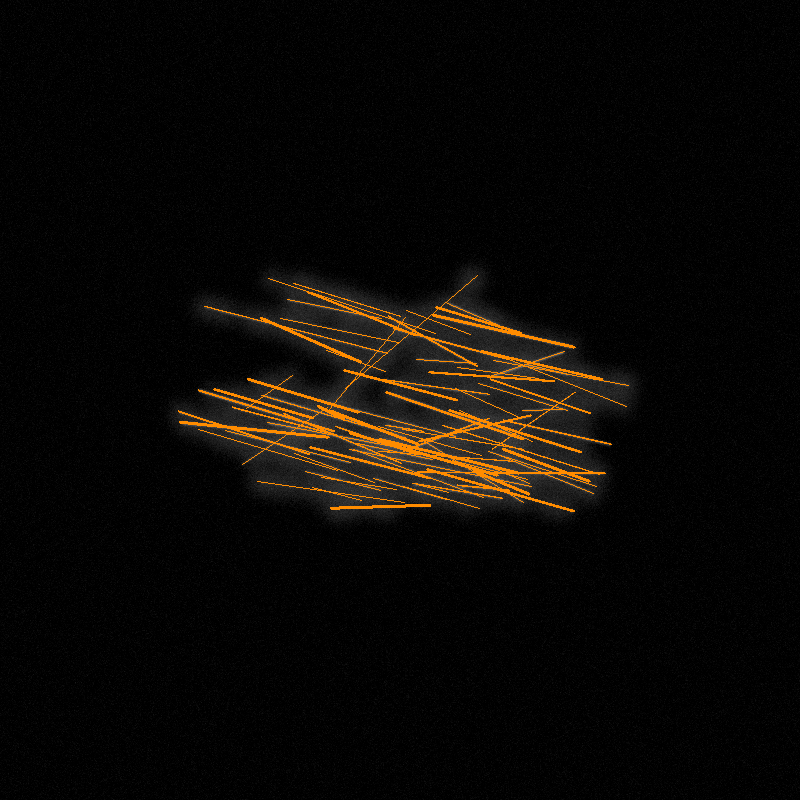

Supplement: S1 File — This comprises the FilamentSensor, the benchmark database, the output of the compared programs, several auxiliary scripts, and the evaluation results of the program outputs. (ZIP) [file pone.0126346.s001.zip › supporting_information/ground_truth/simulated/ground_truth_simulated_img/simulated_cell_01_png_filaments.png]

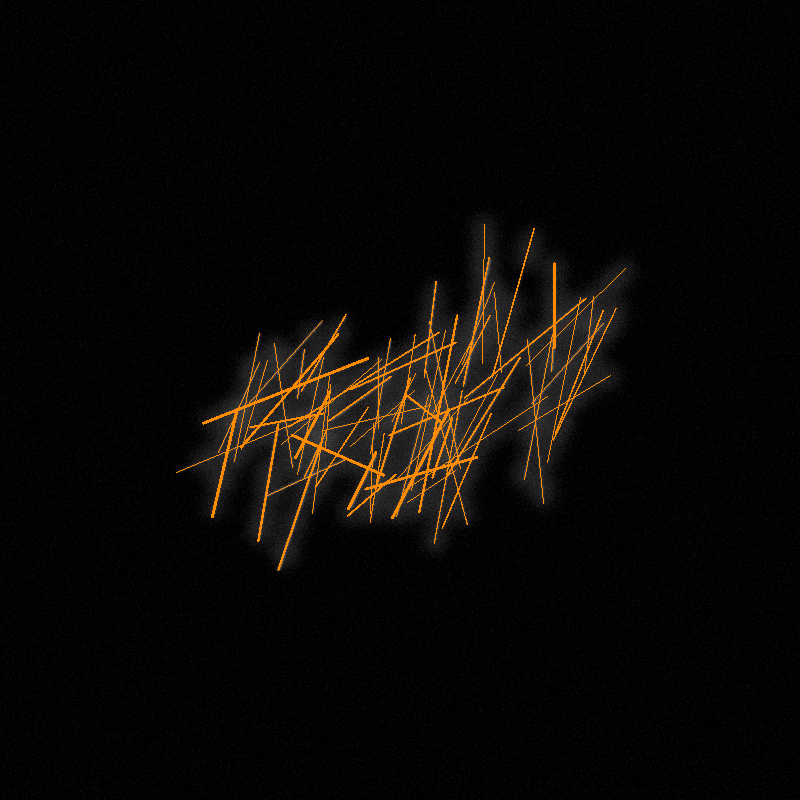

Supplement: S1 File — This comprises the FilamentSensor, the benchmark database, the output of the compared programs, several auxiliary scripts, and the evaluation results of the program outputs. (ZIP) [file pone.0126346.s001.zip › supporting_information/ground_truth/simulated/ground_truth_simulated_img/simulated_cell_02_png_filaments.png]

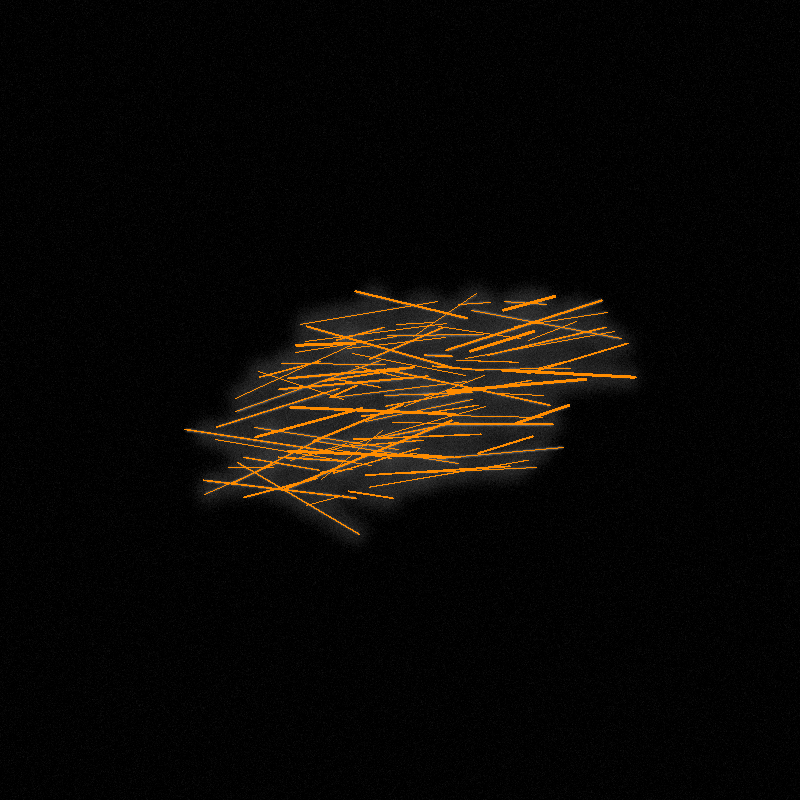

Supplement: S1 File — This comprises the FilamentSensor, the benchmark database, the output of the compared programs, several auxiliary scripts, and the evaluation results of the program outputs. (ZIP) [file pone.0126346.s001.zip › supporting_information/ground_truth/simulated/ground_truth_simulated_img/simulated_cell_10_png_filaments.png]

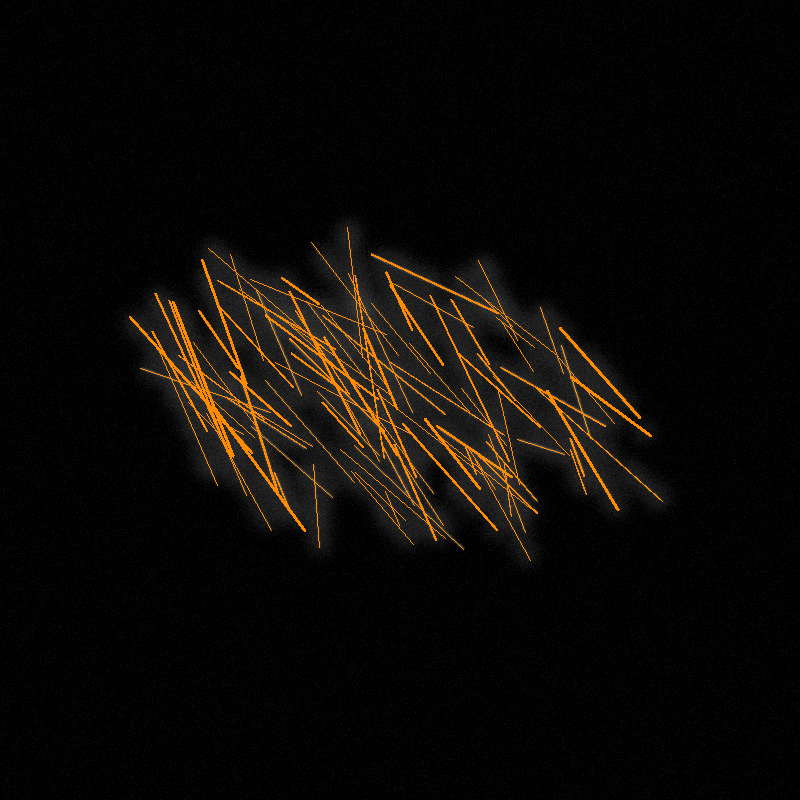

Supplement: S1 File — This comprises the FilamentSensor, the benchmark database, the output of the compared programs, several auxiliary scripts, and the evaluation results of the program outputs. (ZIP) [file pone.0126346.s001.zip › supporting_information/ground_truth/simulated/ground_truth_simulated_img/simulated_cell_03_png_filaments.png]

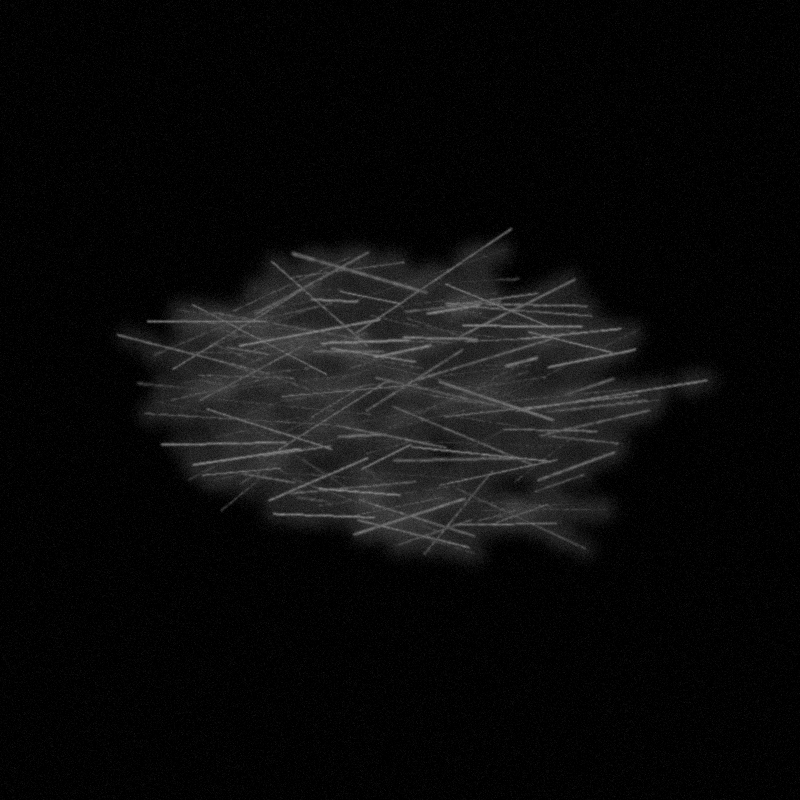

Supplement: S1 File — This comprises the FilamentSensor, the benchmark database, the output of the compared programs, several auxiliary scripts, and the evaluation results of the program outputs. (ZIP) [file pone.0126346.s001.zip › supporting_information/ground_truth/simulated/simulated_cell_09.png]

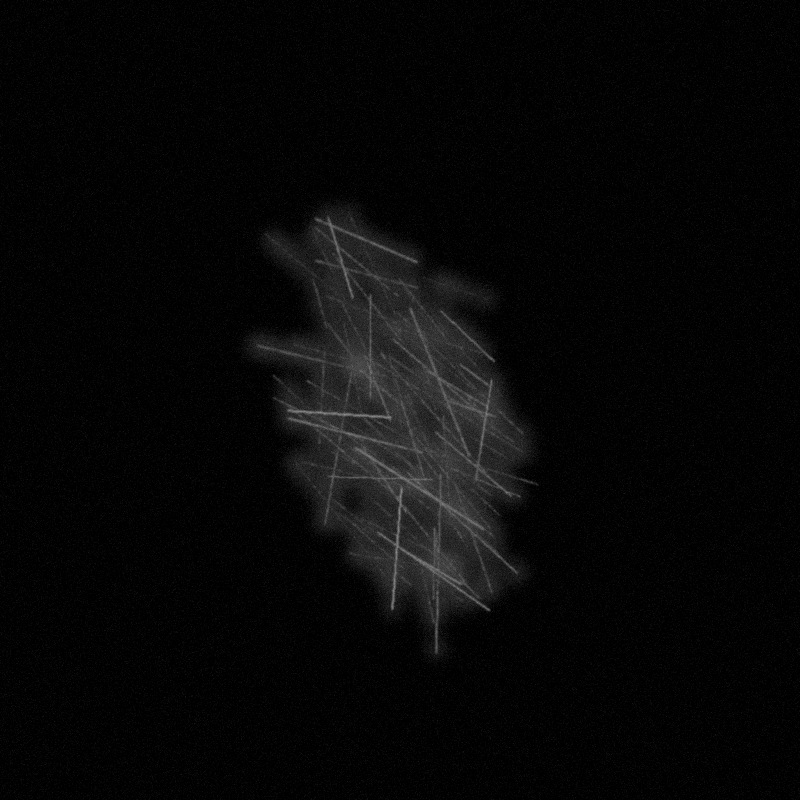

Supplement: S1 File — This comprises the FilamentSensor, the benchmark database, the output of the compared programs, several auxiliary scripts, and the evaluation results of the program outputs. (ZIP) [file pone.0126346.s001.zip › supporting_information/ground_truth/simulated/simulated_cell_04.png]

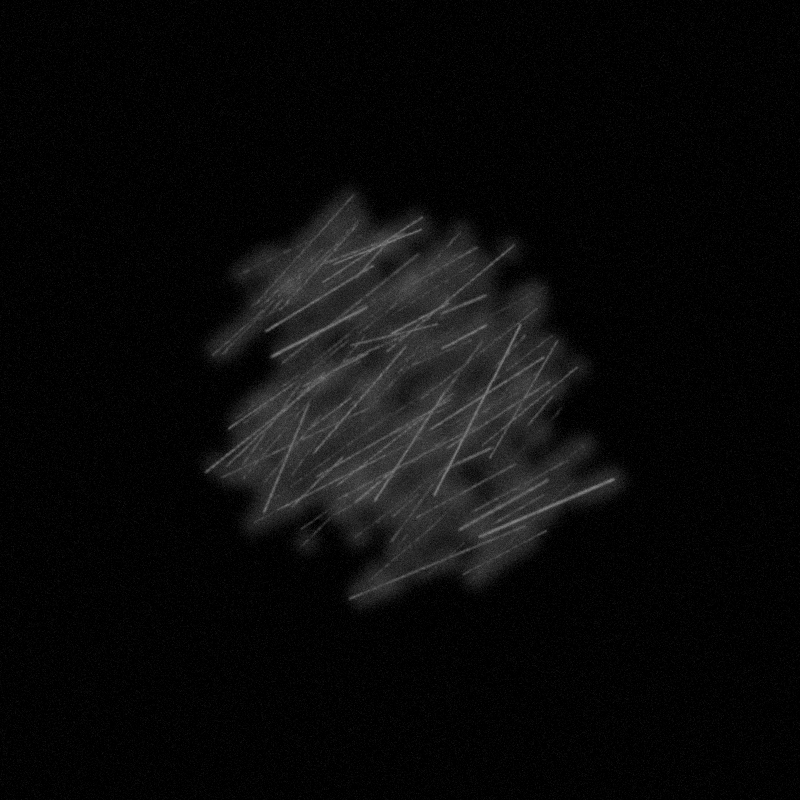

Supplement: S1 File — This comprises the FilamentSensor, the benchmark database, the output of the compared programs, several auxiliary scripts, and the evaluation results of the program outputs. (ZIP) [file pone.0126346.s001.zip › supporting_information/ground_truth/simulated/simulated_cell_05.png]

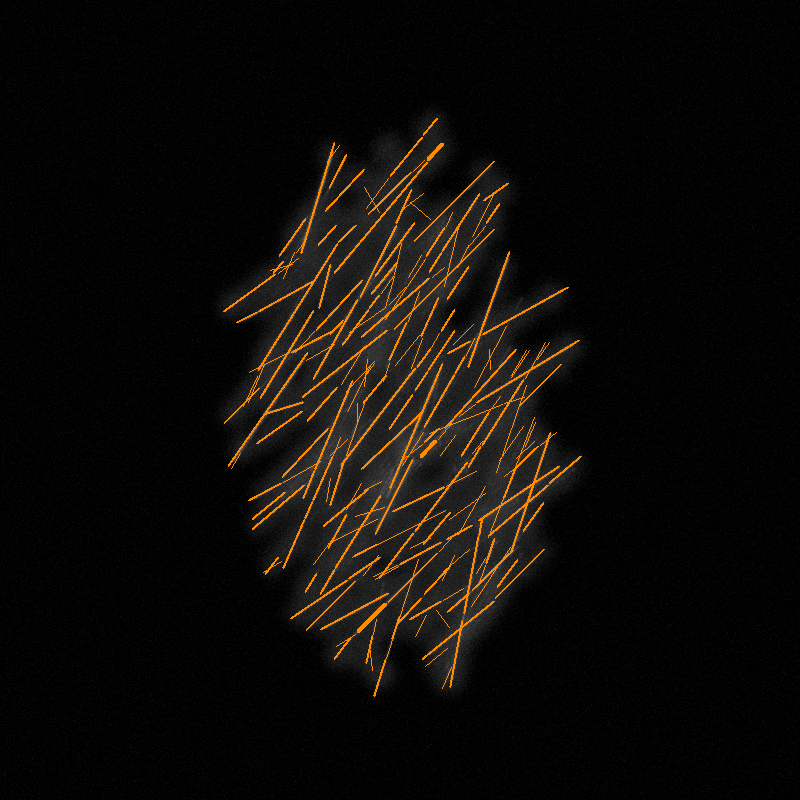

Supplement: S1 File — This comprises the FilamentSensor, the benchmark database, the output of the compared programs, several auxiliary scripts, and the evaluation results of the program outputs. (ZIP) [file pone.0126346.s001.zip › supporting_information/ground_truth/simulated/line_sensor_simulated_img/simulated_cell_07_png_filaments.png]

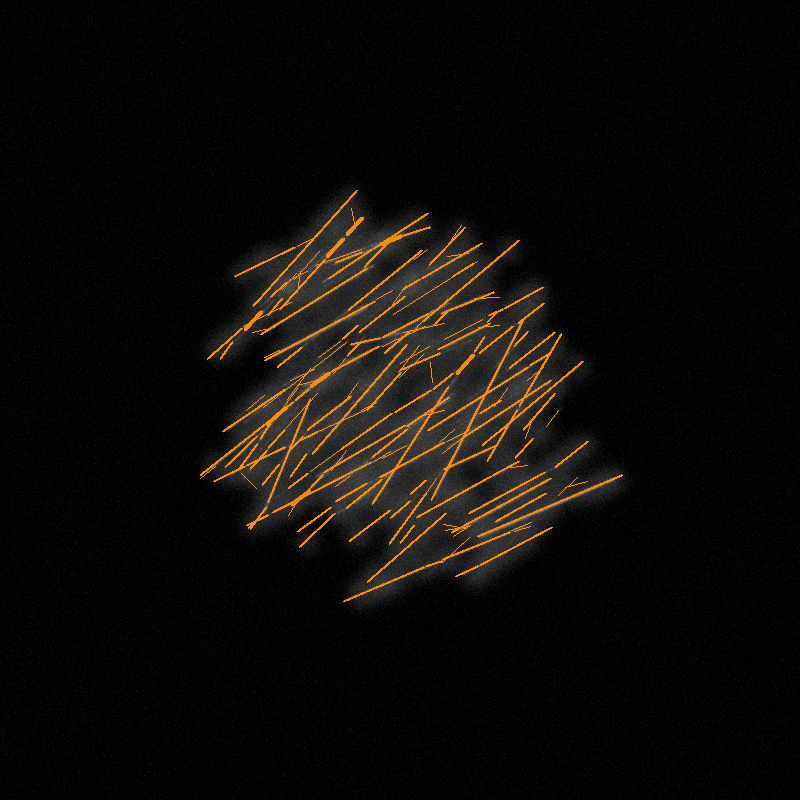

Supplement: S1 File — This comprises the FilamentSensor, the benchmark database, the output of the compared programs, several auxiliary scripts, and the evaluation results of the program outputs. (ZIP) [file pone.0126346.s001.zip › supporting_information/ground_truth/simulated/line_sensor_simulated_img/simulated_cell_05_png_filaments.png]

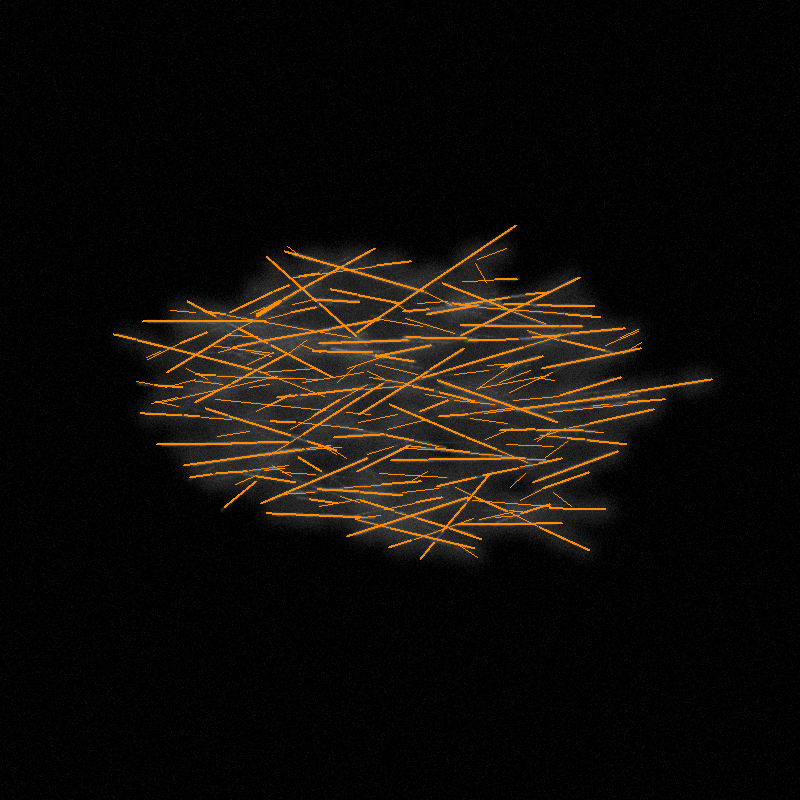

Supplement: S1 File — This comprises the FilamentSensor, the benchmark database, the output of the compared programs, several auxiliary scripts, and the evaluation results of the program outputs. (ZIP) [file pone.0126346.s001.zip › supporting_information/ground_truth/simulated/line_sensor_simulated_img/simulated_cell_09_png_filaments.png]

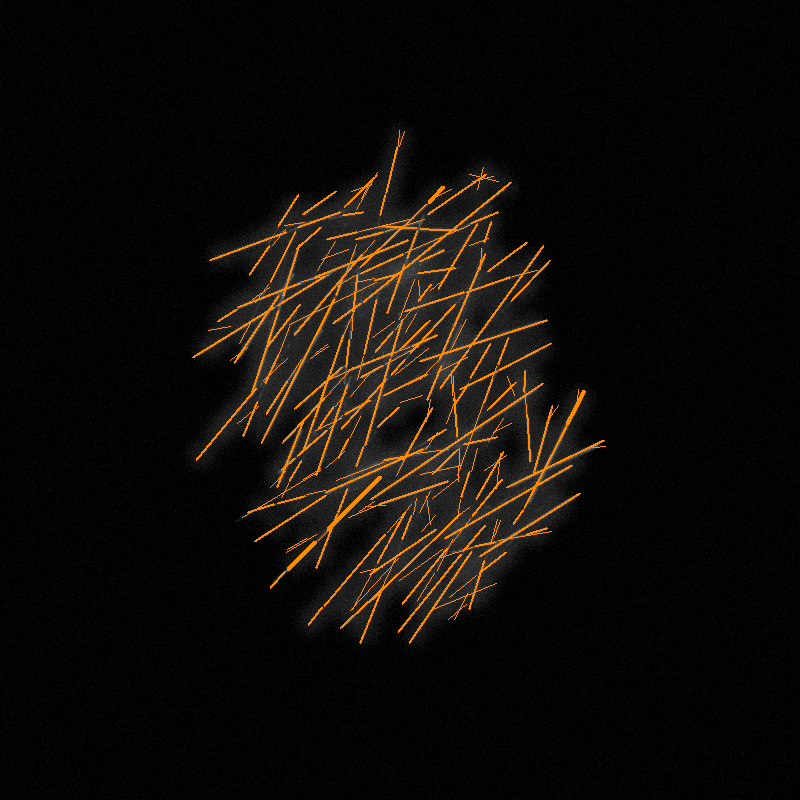

Supplement: S1 File — This comprises the FilamentSensor, the benchmark database, the output of the compared programs, several auxiliary scripts, and the evaluation results of the program outputs. (ZIP) [file pone.0126346.s001.zip › supporting_information/ground_truth/simulated/line_sensor_simulated_img/simulated_cell_06_png_filaments.png]

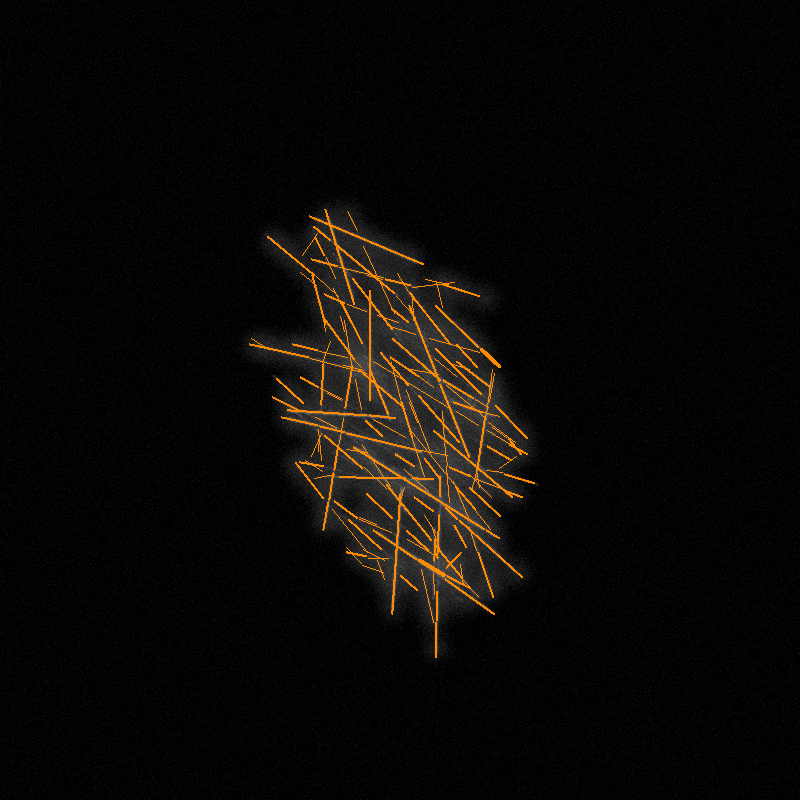

Supplement: S1 File — This comprises the FilamentSensor, the benchmark database, the output of the compared programs, several auxiliary scripts, and the evaluation results of the program outputs. (ZIP) [file pone.0126346.s001.zip › supporting_information/ground_truth/simulated/line_sensor_simulated_img/simulated_cell_04_png_filaments.png]

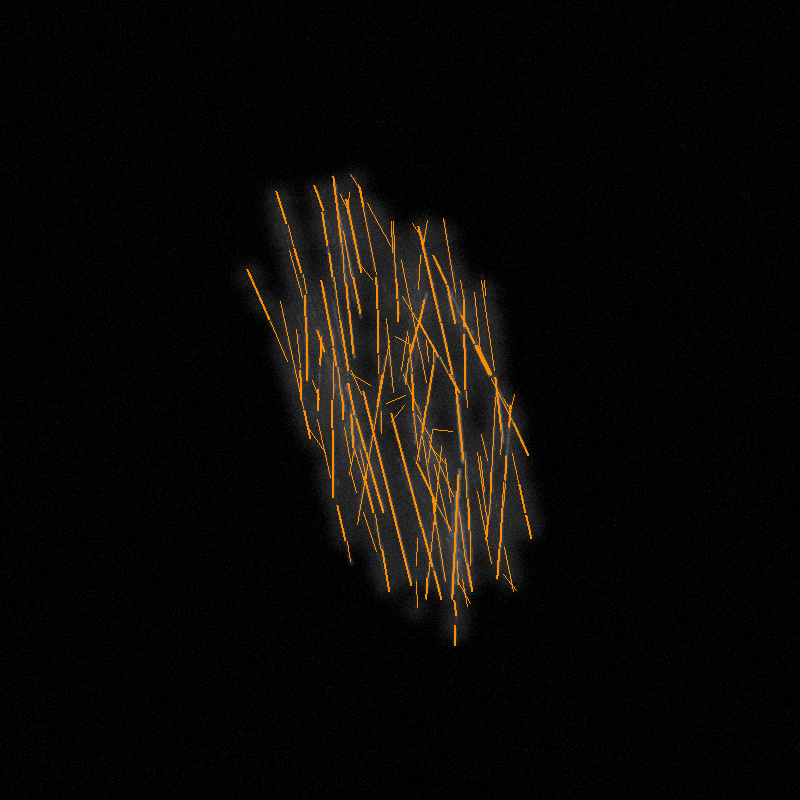

Supplement: S1 File — This comprises the FilamentSensor, the benchmark database, the output of the compared programs, several auxiliary scripts, and the evaluation results of the program outputs. (ZIP) [file pone.0126346.s001.zip › supporting_information/ground_truth/simulated/line_sensor_simulated_img/simulated_cell_08_png_filaments.png]

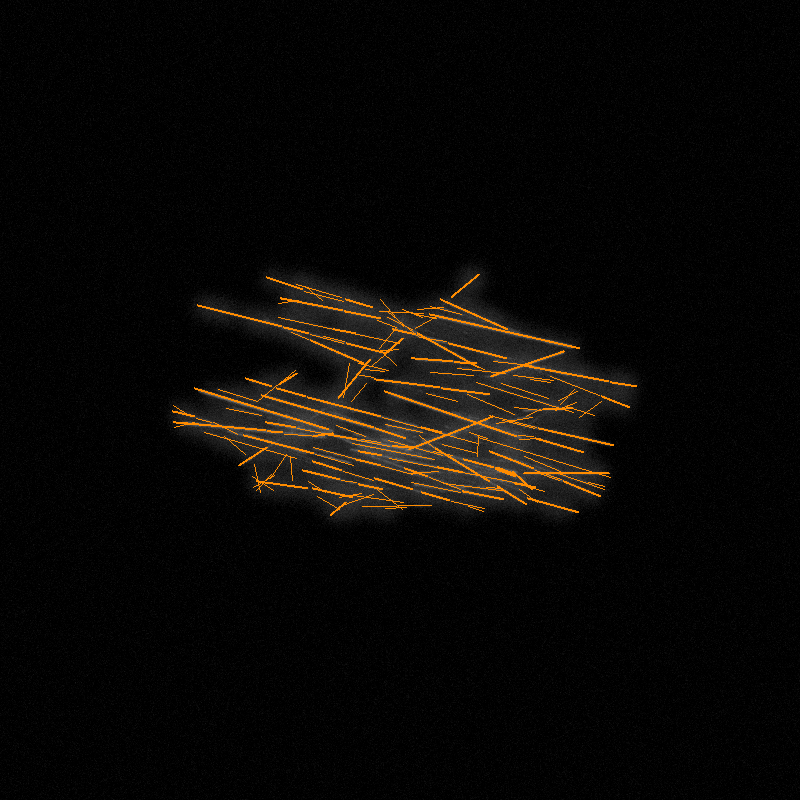

Supplement: S1 File — This comprises the FilamentSensor, the benchmark database, the output of the compared programs, several auxiliary scripts, and the evaluation results of the program outputs. (ZIP) [file pone.0126346.s001.zip › supporting_information/ground_truth/simulated/line_sensor_simulated_img/simulated_cell_01_png_filaments.png]

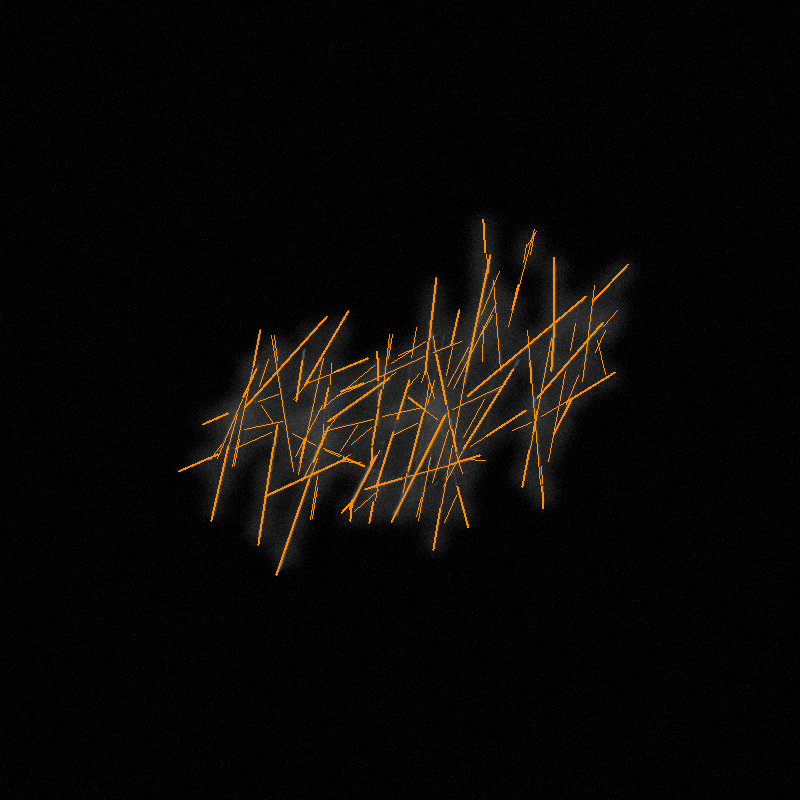

Supplement: S1 File — This comprises the FilamentSensor, the benchmark database, the output of the compared programs, several auxiliary scripts, and the evaluation results of the program outputs. (ZIP) [file pone.0126346.s001.zip › supporting_information/ground_truth/simulated/line_sensor_simulated_img/simulated_cell_02_png_filaments.png]

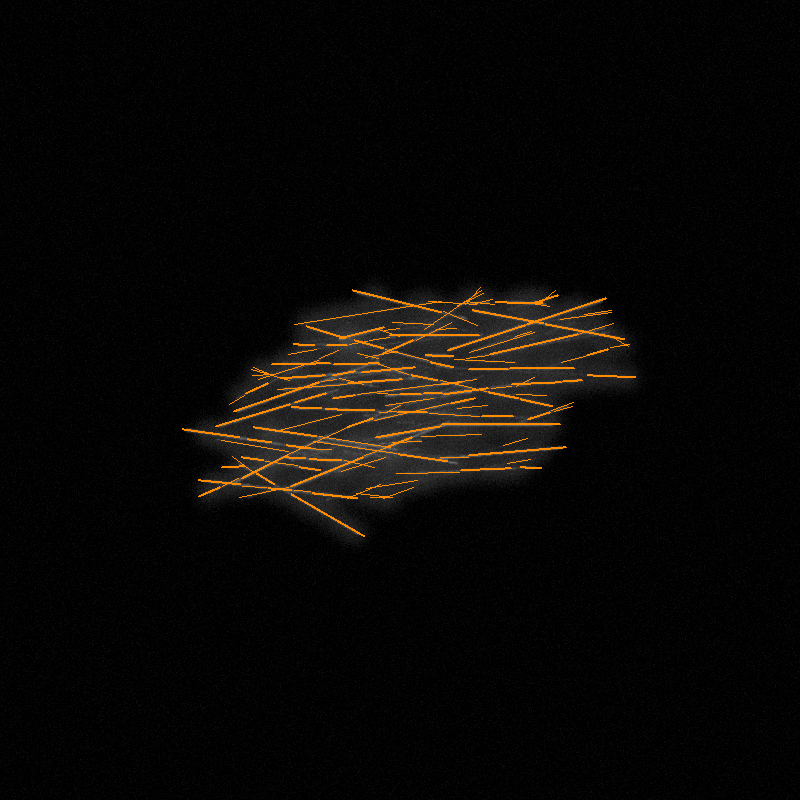

Supplement: S1 File — This comprises the FilamentSensor, the benchmark database, the output of the compared programs, several auxiliary scripts, and the evaluation results of the program outputs. (ZIP) [file pone.0126346.s001.zip › supporting_information/ground_truth/simulated/line_sensor_simulated_img/simulated_cell_10_png_filaments.png]

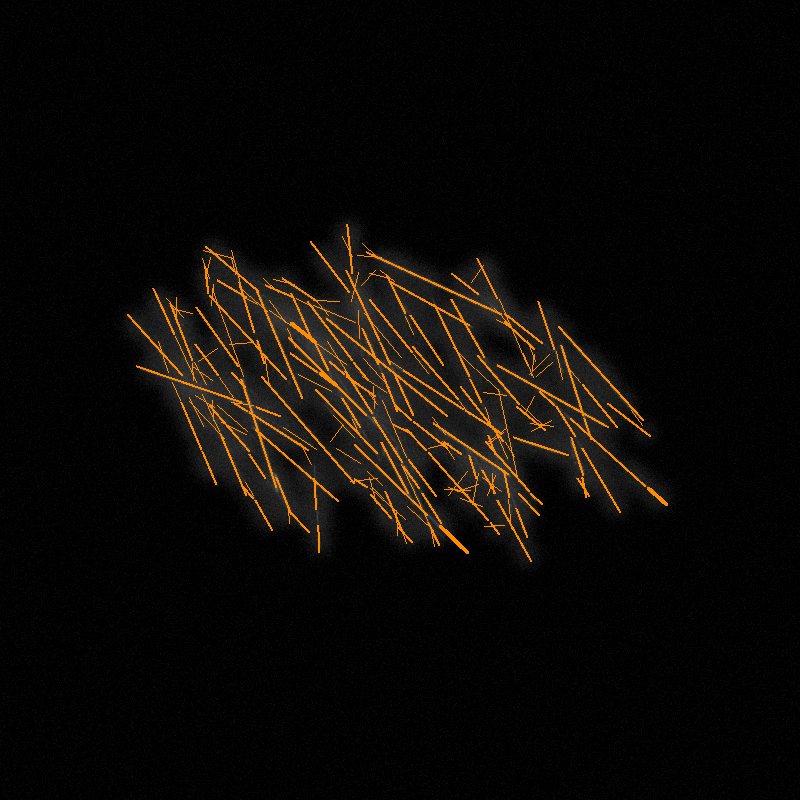

Supplement: S1 File — This comprises the FilamentSensor, the benchmark database, the output of the compared programs, several auxiliary scripts, and the evaluation results of the program outputs. (ZIP) [file pone.0126346.s001.zip › supporting_information/ground_truth/simulated/line_sensor_simulated_img/simulated_cell_03_png_filaments.png]

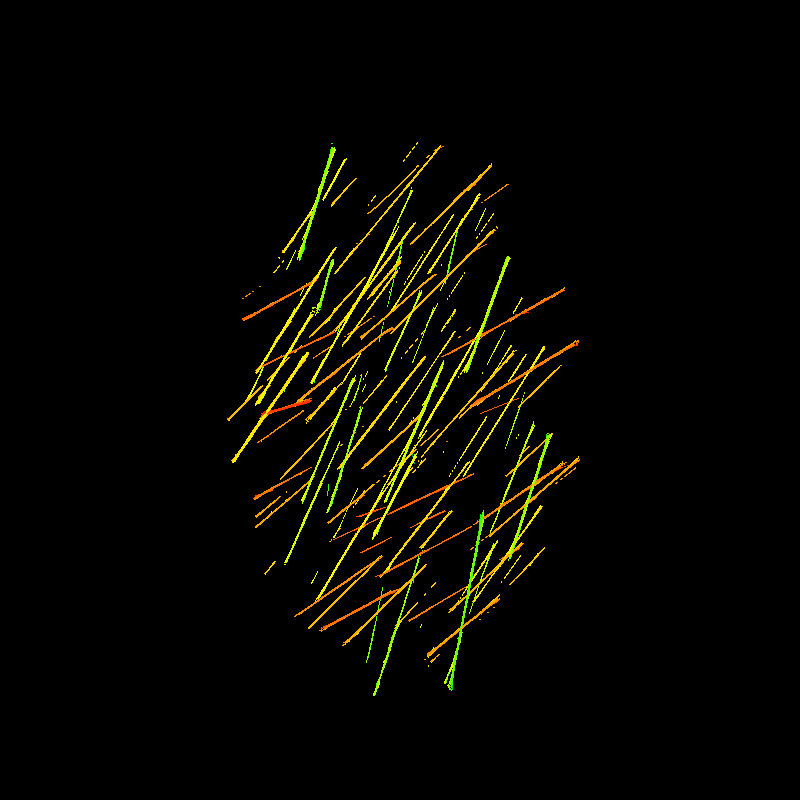

Supplement: S1 File — This comprises the FilamentSensor, the benchmark database, the output of the compared programs, several auxiliary scripts, and the evaluation results of the program outputs. (ZIP) [file pone.0126346.s001.zip › supporting_information/ground_truth/simulated/eLoG_simulated_img/simulated_cell_07_png_filaments.png]

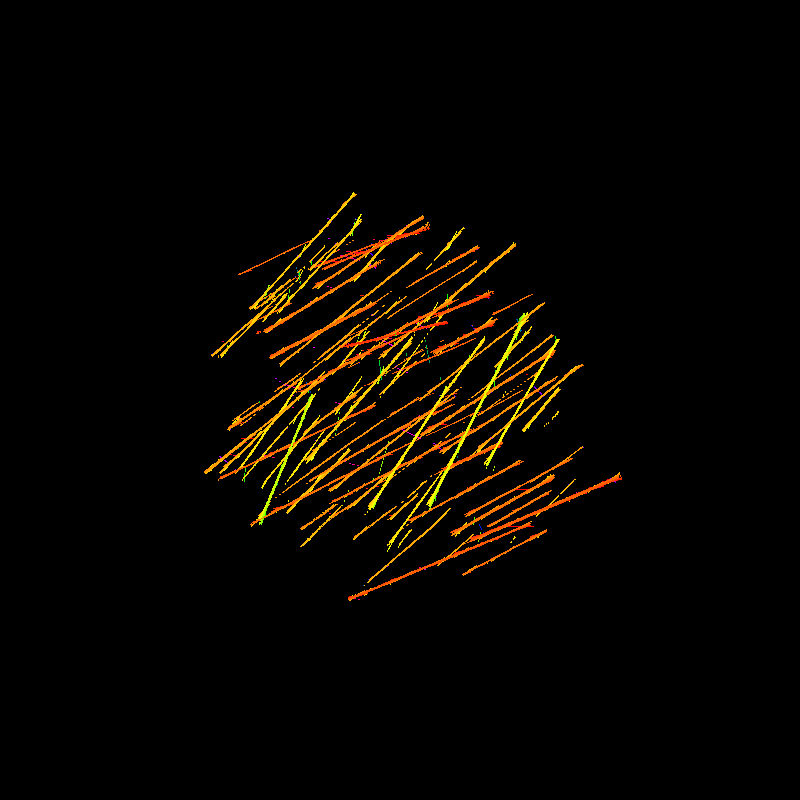

Supplement: S1 File — This comprises the FilamentSensor, the benchmark database, the output of the compared programs, several auxiliary scripts, and the evaluation results of the program outputs. (ZIP) [file pone.0126346.s001.zip › supporting_information/ground_truth/simulated/eLoG_simulated_img/simulated_cell_05_png_filaments.png]

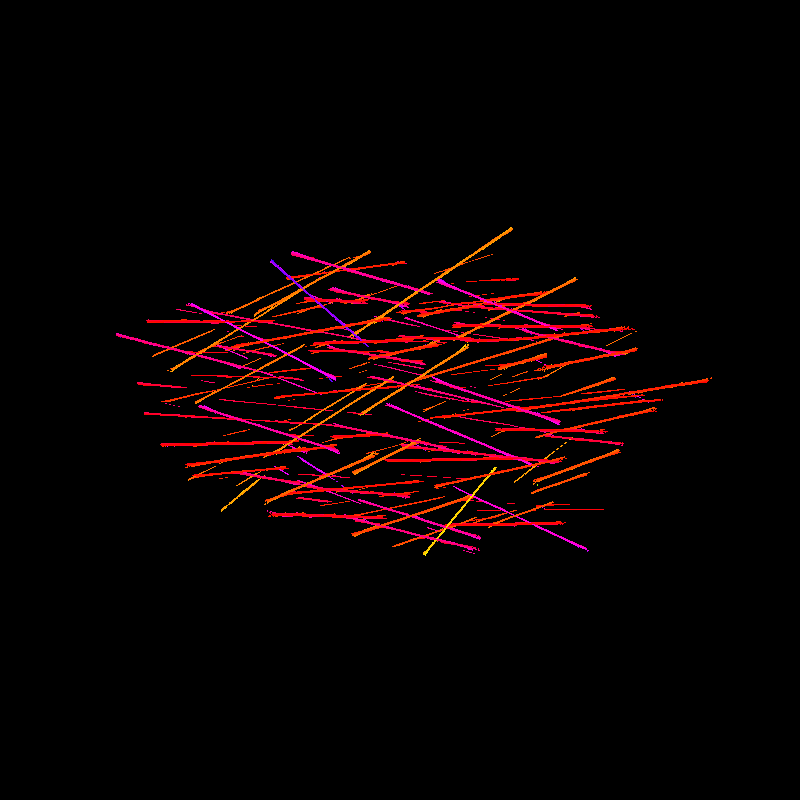

Supplement: S1 File — This comprises the FilamentSensor, the benchmark database, the output of the compared programs, several auxiliary scripts, and the evaluation results of the program outputs. (ZIP) [file pone.0126346.s001.zip › supporting_information/ground_truth/simulated/eLoG_simulated_img/simulated_cell_09_png_filaments.png]

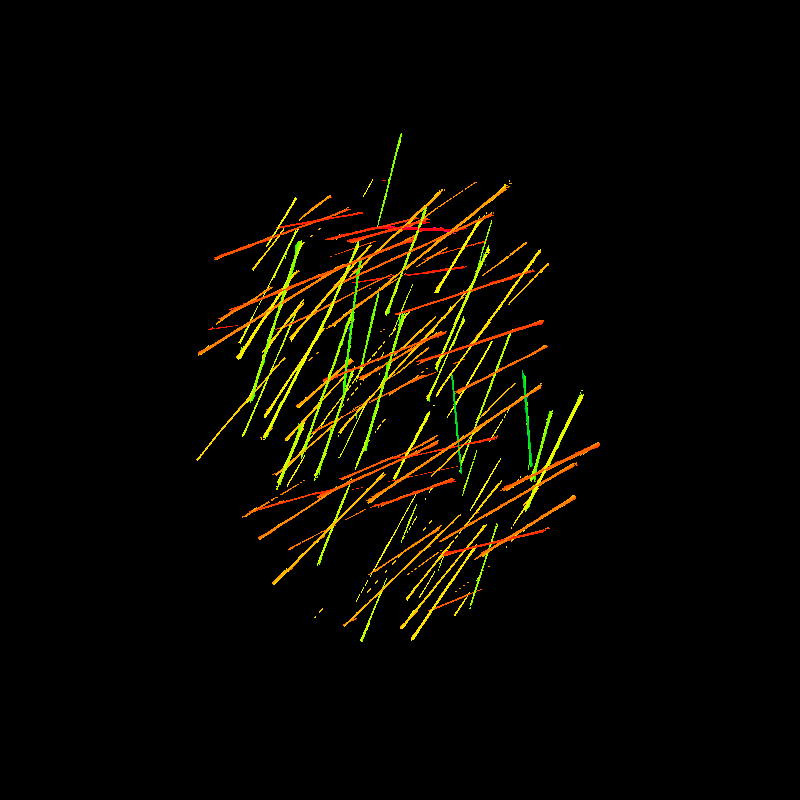

Supplement: S1 File — This comprises the FilamentSensor, the benchmark database, the output of the compared programs, several auxiliary scripts, and the evaluation results of the program outputs. (ZIP) [file pone.0126346.s001.zip › supporting_information/ground_truth/simulated/eLoG_simulated_img/simulated_cell_06_png_filaments.png]

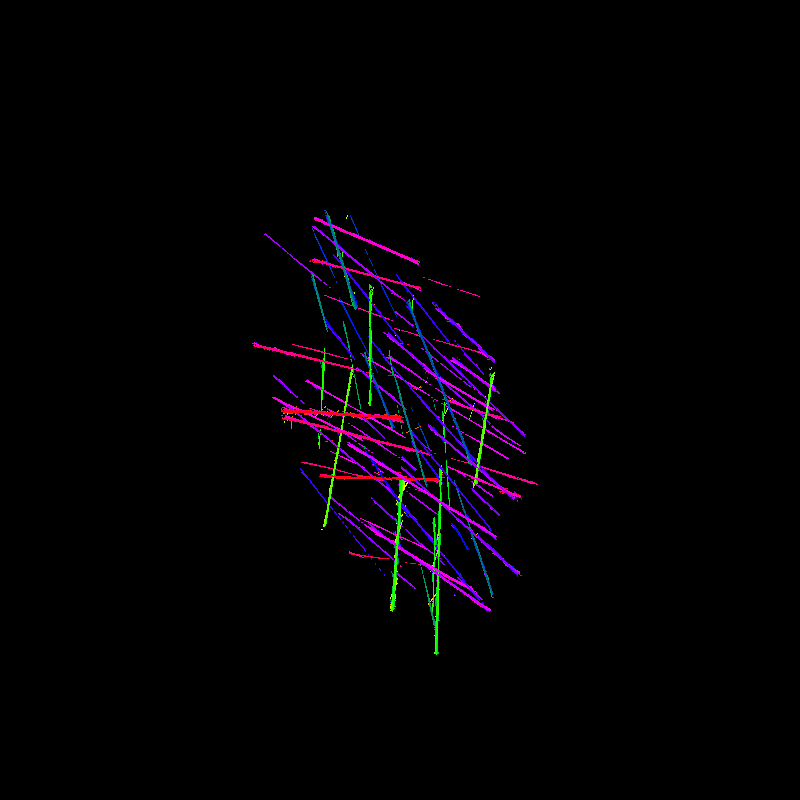

Supplement: S1 File — This comprises the FilamentSensor, the benchmark database, the output of the compared programs, several auxiliary scripts, and the evaluation results of the program outputs. (ZIP) [file pone.0126346.s001.zip › supporting_information/ground_truth/simulated/eLoG_simulated_img/simulated_cell_04_png_filaments.png]

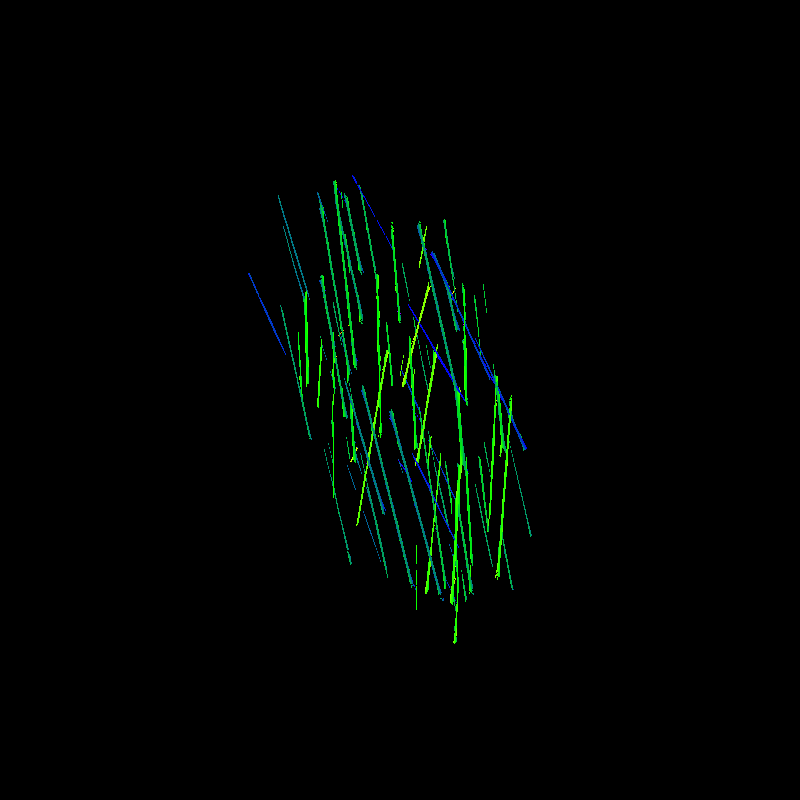

Supplement: S1 File — This comprises the FilamentSensor, the benchmark database, the output of the compared programs, several auxiliary scripts, and the evaluation results of the program outputs. (ZIP) [file pone.0126346.s001.zip › supporting_information/ground_truth/simulated/eLoG_simulated_img/simulated_cell_08_png_filaments.png]

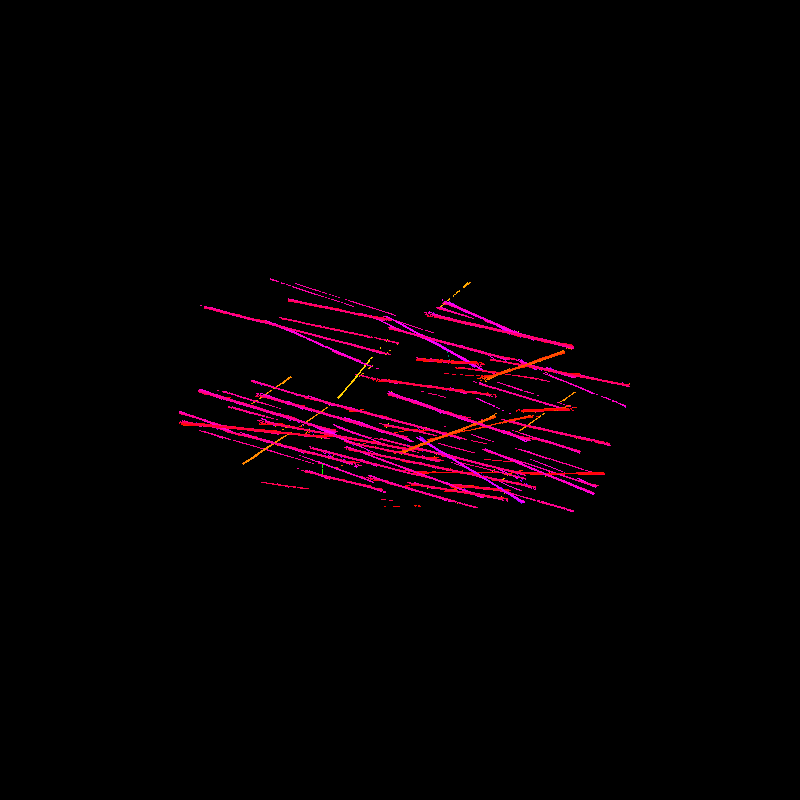

Supplement: S1 File — This comprises the FilamentSensor, the benchmark database, the output of the compared programs, several auxiliary scripts, and the evaluation results of the program outputs. (ZIP) [file pone.0126346.s001.zip › supporting_information/ground_truth/simulated/eLoG_simulated_img/simulated_cell_01_png_filaments.png]

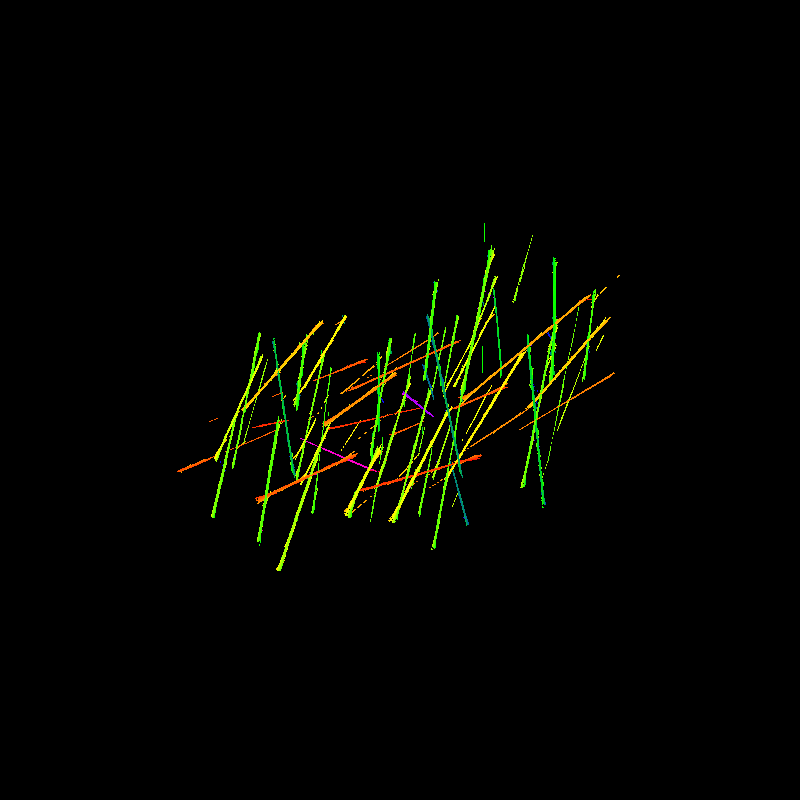

Supplement: S1 File — This comprises the FilamentSensor, the benchmark database, the output of the compared programs, several auxiliary scripts, and the evaluation results of the program outputs. (ZIP) [file pone.0126346.s001.zip › supporting_information/ground_truth/simulated/eLoG_simulated_img/simulated_cell_02_png_filaments.png]

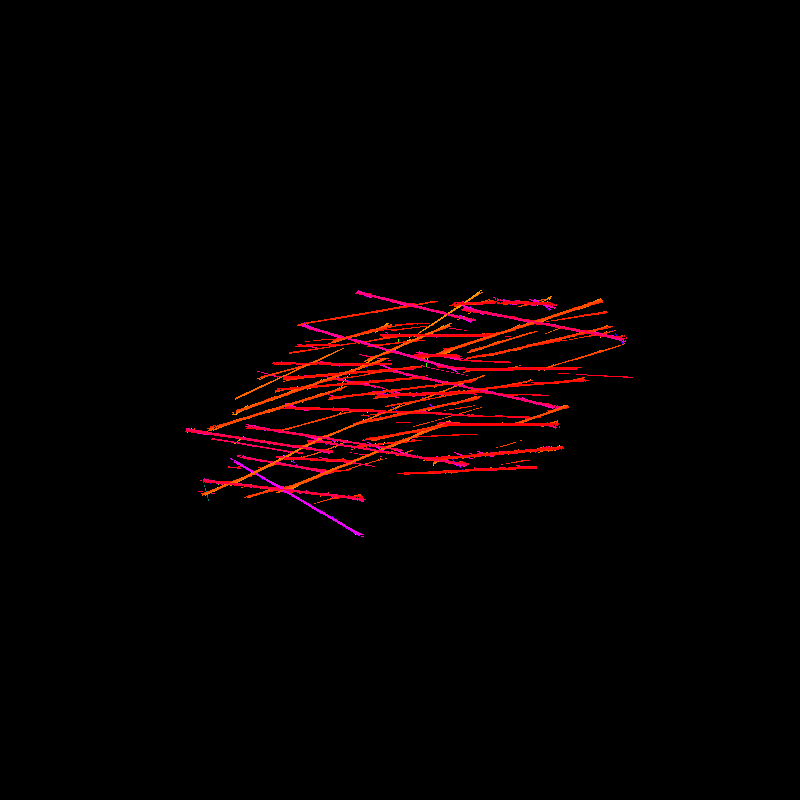

Supplement: S1 File — This comprises the FilamentSensor, the benchmark database, the output of the compared programs, several auxiliary scripts, and the evaluation results of the program outputs. (ZIP) [file pone.0126346.s001.zip › supporting_information/ground_truth/simulated/eLoG_simulated_img/simulated_cell_10_png_filaments.png]

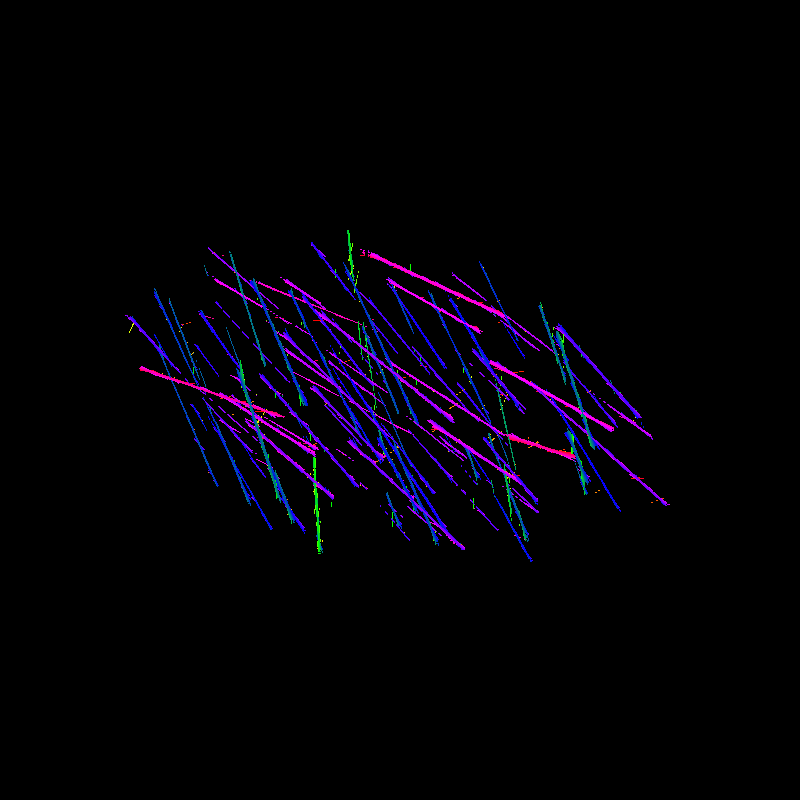

Supplement: S1 File — This comprises the FilamentSensor, the benchmark database, the output of the compared programs, several auxiliary scripts, and the evaluation results of the program outputs. (ZIP) [file pone.0126346.s001.zip › supporting_information/ground_truth/simulated/eLoG_simulated_img/simulated_cell_03_png_filaments.png]

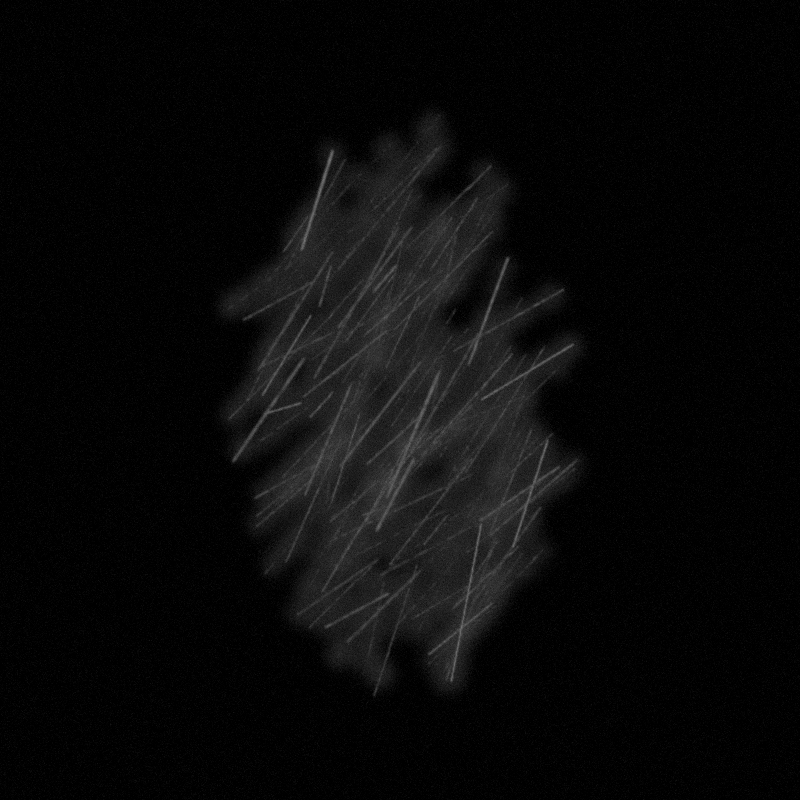

Supplement: S1 File — This comprises the FilamentSensor, the benchmark database, the output of the compared programs, several auxiliary scripts, and the evaluation results of the program outputs. (ZIP) [file pone.0126346.s001.zip › supporting_information/ground_truth/simulated/simulated_cell_07.png]

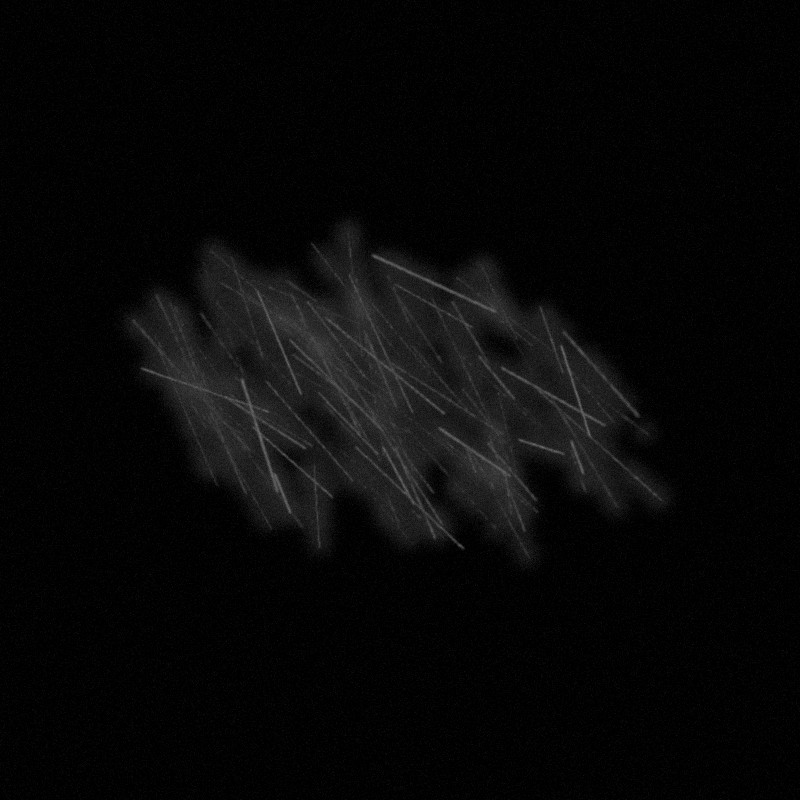

Supplement: S1 File — This comprises the FilamentSensor, the benchmark database, the output of the compared programs, several auxiliary scripts, and the evaluation results of the program outputs. (ZIP) [file pone.0126346.s001.zip › supporting_information/ground_truth/simulated/simulated_cell_03.png]

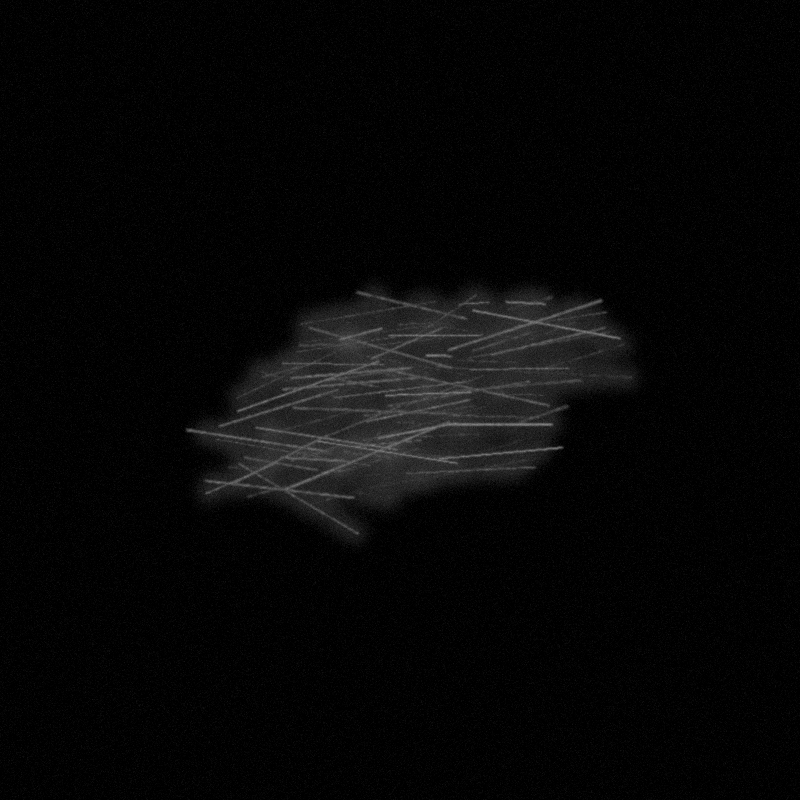

Supplement: S1 File — This comprises the FilamentSensor, the benchmark database, the output of the compared programs, several auxiliary scripts, and the evaluation results of the program outputs. (ZIP) [file pone.0126346.s001.zip › supporting_information/ground_truth/simulated/simulated_cell_10.png]

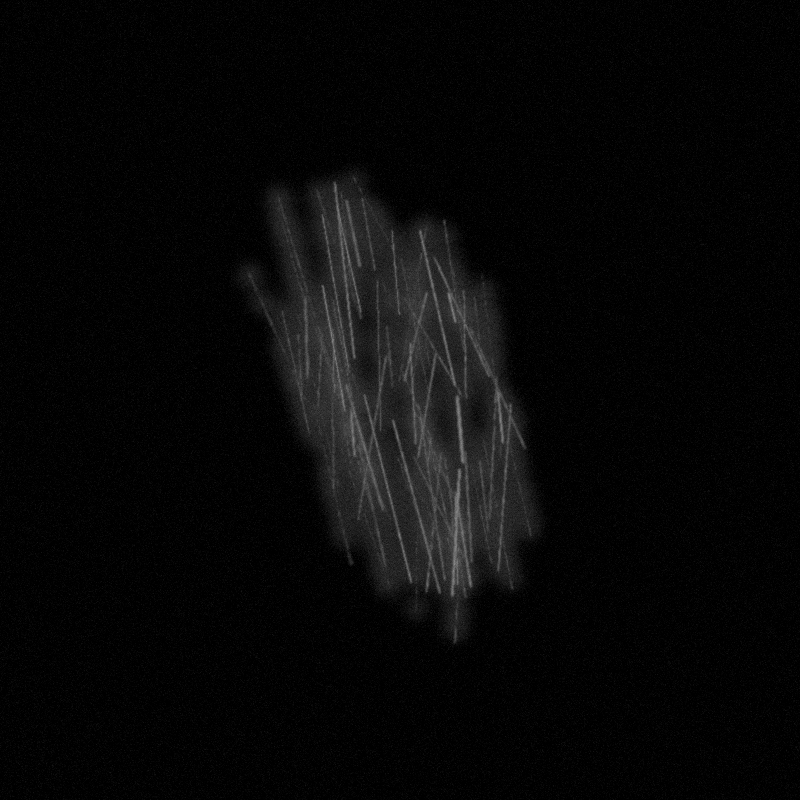

Supplement: S1 File — This comprises the FilamentSensor, the benchmark database, the output of the compared programs, several auxiliary scripts, and the evaluation results of the program outputs. (ZIP) [file pone.0126346.s001.zip › supporting_information/ground_truth/simulated/simulated_cell_08.png]

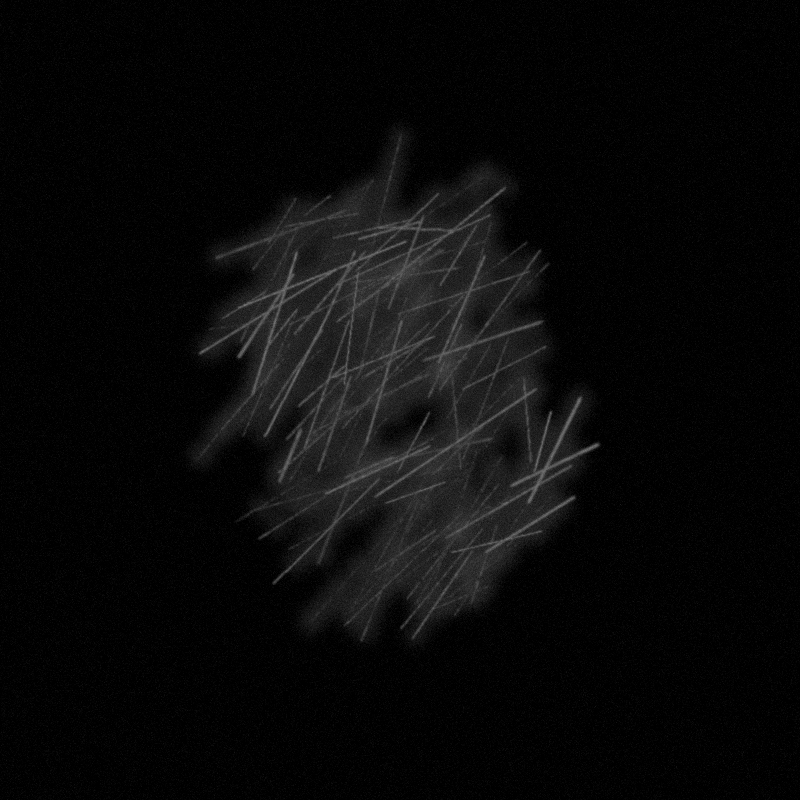

Supplement: S1 File — This comprises the FilamentSensor, the benchmark database, the output of the compared programs, several auxiliary scripts, and the evaluation results of the program outputs. (ZIP) [file pone.0126346.s001.zip › supporting_information/ground_truth/simulated/simulated_cell_06.png]

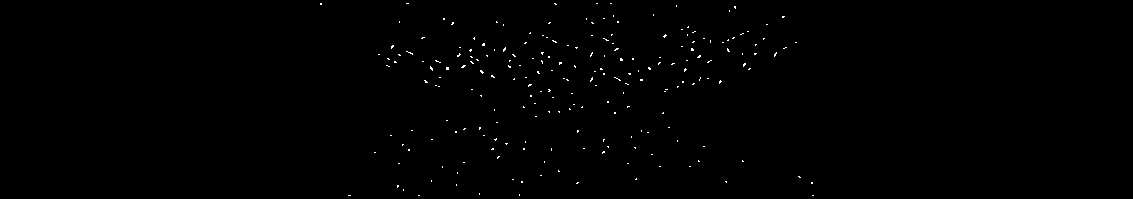

Supplement: S1 File — This comprises the FilamentSensor, the benchmark database, the output of the compared programs, several auxiliary scripts, and the evaluation results of the program outputs. (ZIP) [file pone.0126346.s001.zip › supporting_information/ground_truth/simulated/hough_simulated_img/simulated_cell_07_png_filaments.png]

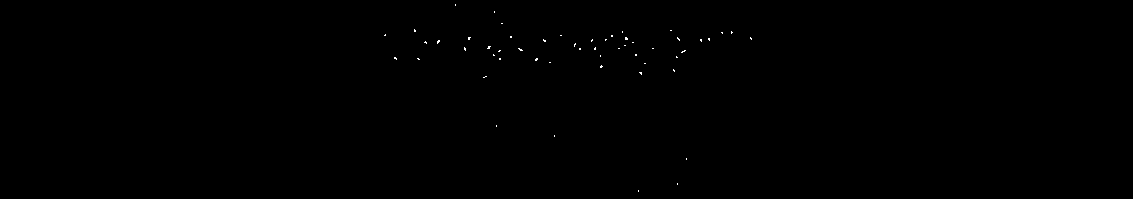

Supplement: S1 File — This comprises the FilamentSensor, the benchmark database, the output of the compared programs, several auxiliary scripts, and the evaluation results of the program outputs. (ZIP) [file pone.0126346.s001.zip › supporting_information/ground_truth/simulated/hough_simulated_img/simulated_cell_05_png_filaments.png]

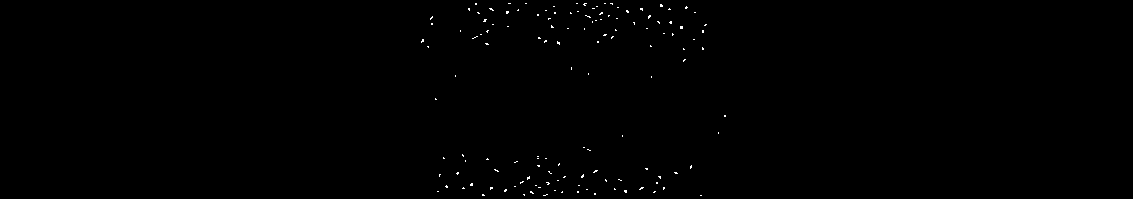

Supplement: S1 File — This comprises the FilamentSensor, the benchmark database, the output of the compared programs, several auxiliary scripts, and the evaluation results of the program outputs. (ZIP) [file pone.0126346.s001.zip › supporting_information/ground_truth/simulated/hough_simulated_img/simulated_cell_09_png_filaments.png]

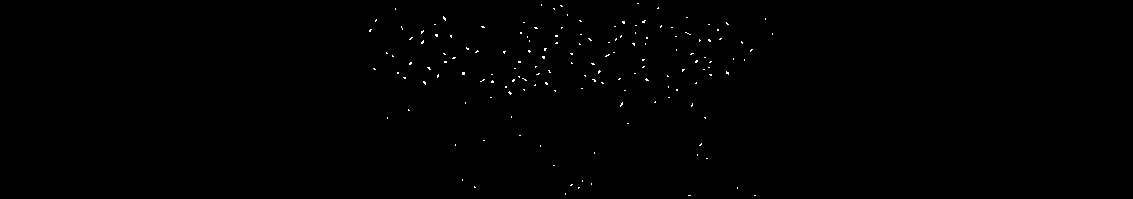

Supplement: S1 File — This comprises the FilamentSensor, the benchmark database, the output of the compared programs, several auxiliary scripts, and the evaluation results of the program outputs. (ZIP) [file pone.0126346.s001.zip › supporting_information/ground_truth/simulated/hough_simulated_img/simulated_cell_06_png_filaments.png]

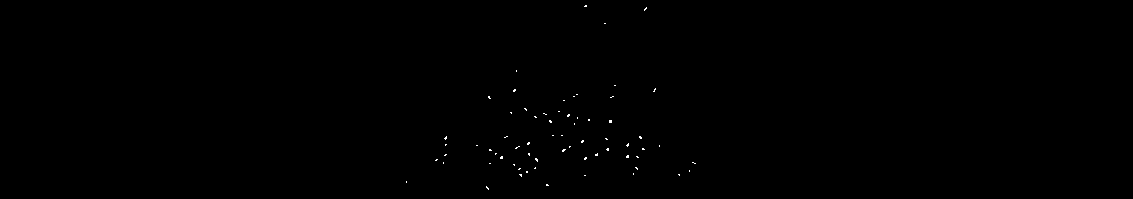

Supplement: S1 File — This comprises the FilamentSensor, the benchmark database, the output of the compared programs, several auxiliary scripts, and the evaluation results of the program outputs. (ZIP) [file pone.0126346.s001.zip › supporting_information/ground_truth/simulated/hough_simulated_img/simulated_cell_04_png_filaments.png]

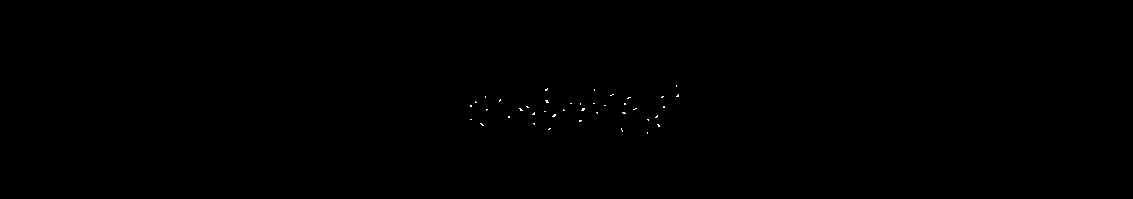

Supplement: S1 File — This comprises the FilamentSensor, the benchmark database, the output of the compared programs, several auxiliary scripts, and the evaluation results of the program outputs. (ZIP) [file pone.0126346.s001.zip › supporting_information/ground_truth/simulated/hough_simulated_img/simulated_cell_08_png_filaments.png]

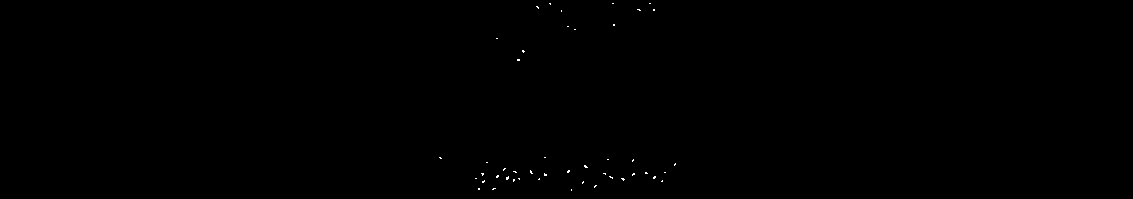

Supplement: S1 File — This comprises the FilamentSensor, the benchmark database, the output of the compared programs, several auxiliary scripts, and the evaluation results of the program outputs. (ZIP) [file pone.0126346.s001.zip › supporting_information/ground_truth/simulated/hough_simulated_img/simulated_cell_01_png_filaments.png]

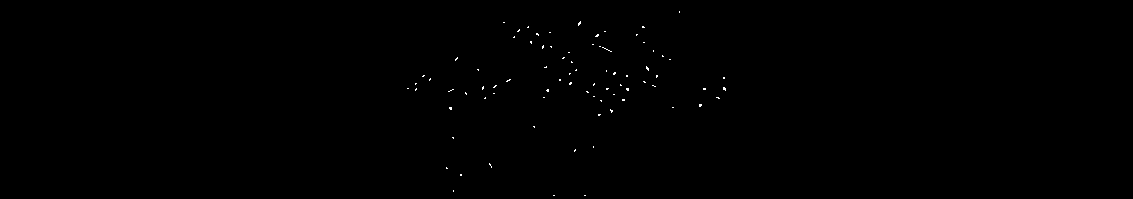

Supplement: S1 File — This comprises the FilamentSensor, the benchmark database, the output of the compared programs, several auxiliary scripts, and the evaluation results of the program outputs. (ZIP) [file pone.0126346.s001.zip › supporting_information/ground_truth/simulated/hough_simulated_img/simulated_cell_02_png_filaments.png]

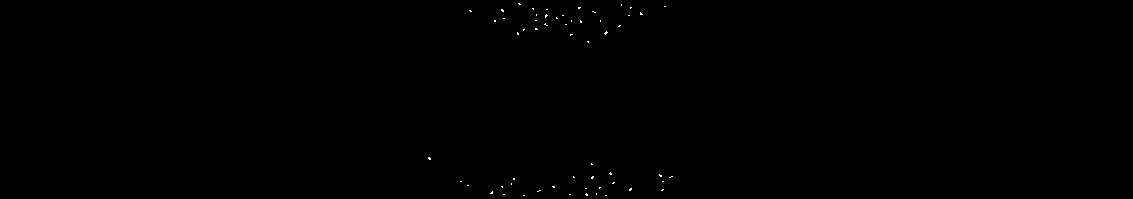

Supplement: S1 File — This comprises the FilamentSensor, the benchmark database, the output of the compared programs, several auxiliary scripts, and the evaluation results of the program outputs. (ZIP) [file pone.0126346.s001.zip › supporting_information/ground_truth/simulated/hough_simulated_img/simulated_cell_10_png_filaments.png]

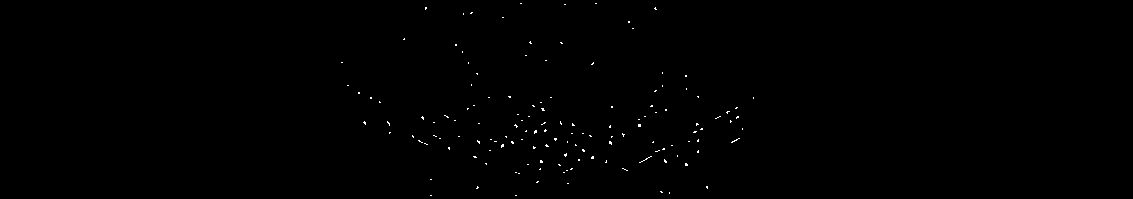

Supplement: S1 File — This comprises the FilamentSensor, the benchmark database, the output of the compared programs, several auxiliary scripts, and the evaluation results of the program outputs. (ZIP) [file pone.0126346.s001.zip › supporting_information/ground_truth/simulated/hough_simulated_img/simulated_cell_03_png_filaments.png]

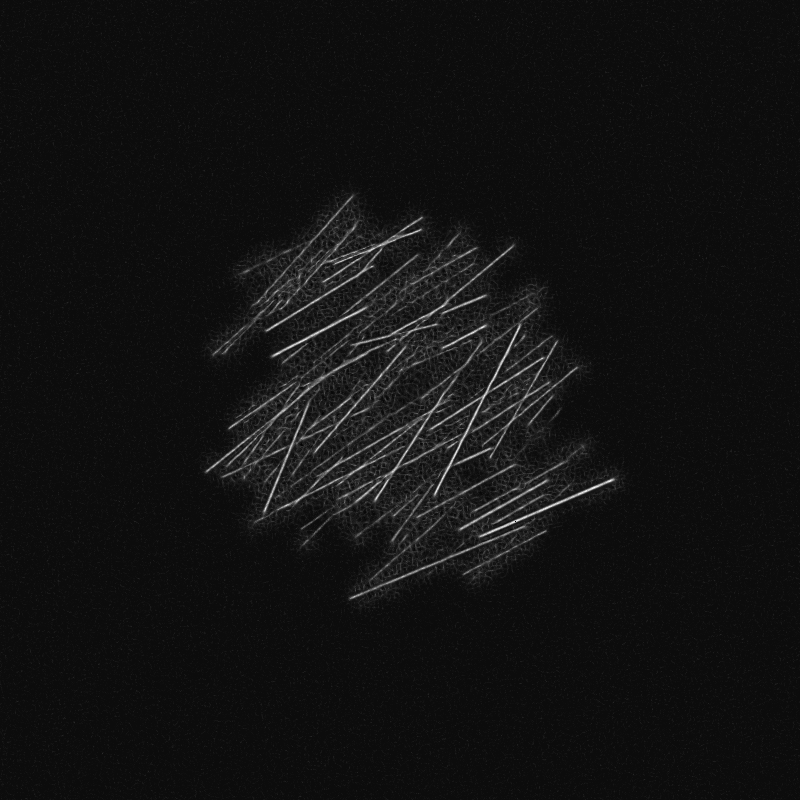

Supplement: S1 File — This comprises the FilamentSensor, the benchmark database, the output of the compared programs, several auxiliary scripts, and the evaluation results of the program outputs. (ZIP) [file pone.0126346.s001.zip › supporting_information/ground_truth/simulated/CID/simulated_cell_05_xcorr.png]

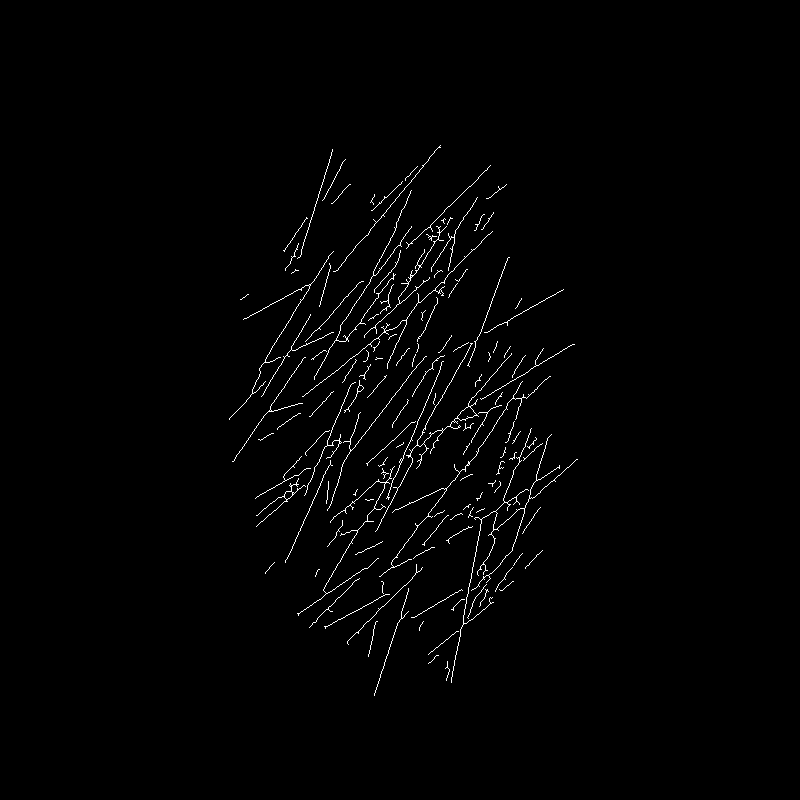

Supplement: S1 File — This comprises the FilamentSensor, the benchmark database, the output of the compared programs, several auxiliary scripts, and the evaluation results of the program outputs. (ZIP) [file pone.0126346.s001.zip › supporting_information/ground_truth/simulated/CID/simulated_cell_07_bin_img.png]

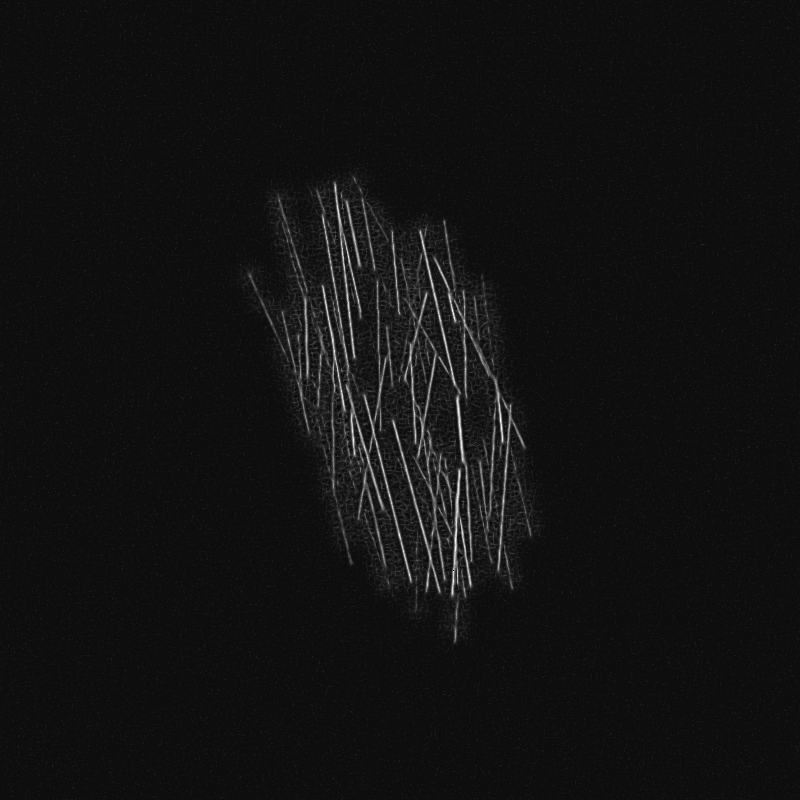

Supplement: S1 File — This comprises the FilamentSensor, the benchmark database, the output of the compared programs, several auxiliary scripts, and the evaluation results of the program outputs. (ZIP) [file pone.0126346.s001.zip › supporting_information/ground_truth/simulated/CID/simulated_cell_08_xcorr.png]

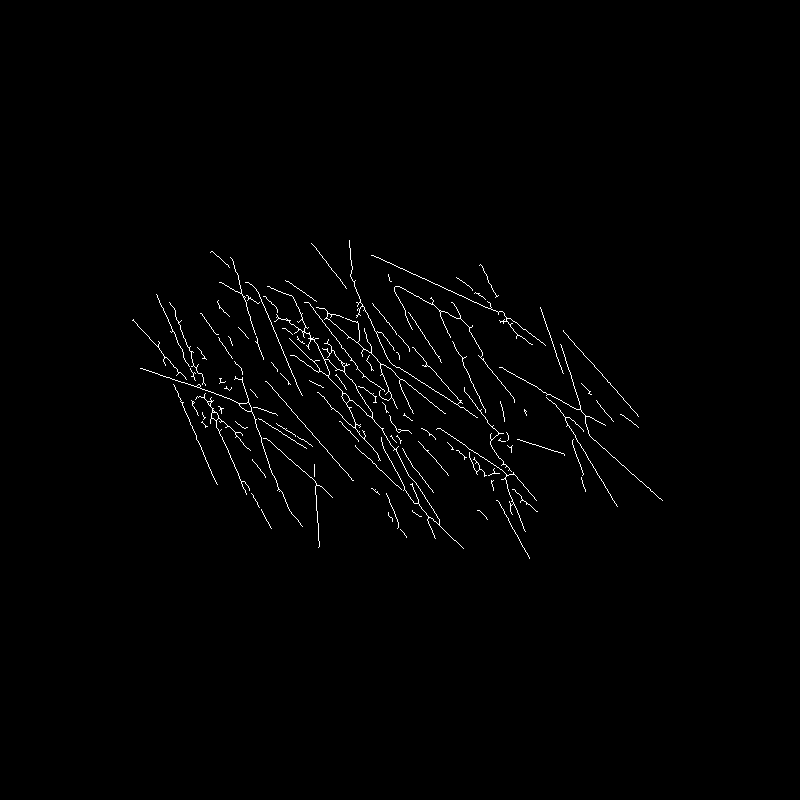

Supplement: S1 File — This comprises the FilamentSensor, the benchmark database, the output of the compared programs, several auxiliary scripts, and the evaluation results of the program outputs. (ZIP) [file pone.0126346.s001.zip › supporting_information/ground_truth/simulated/CID/simulated_cell_03_bin_img.png]

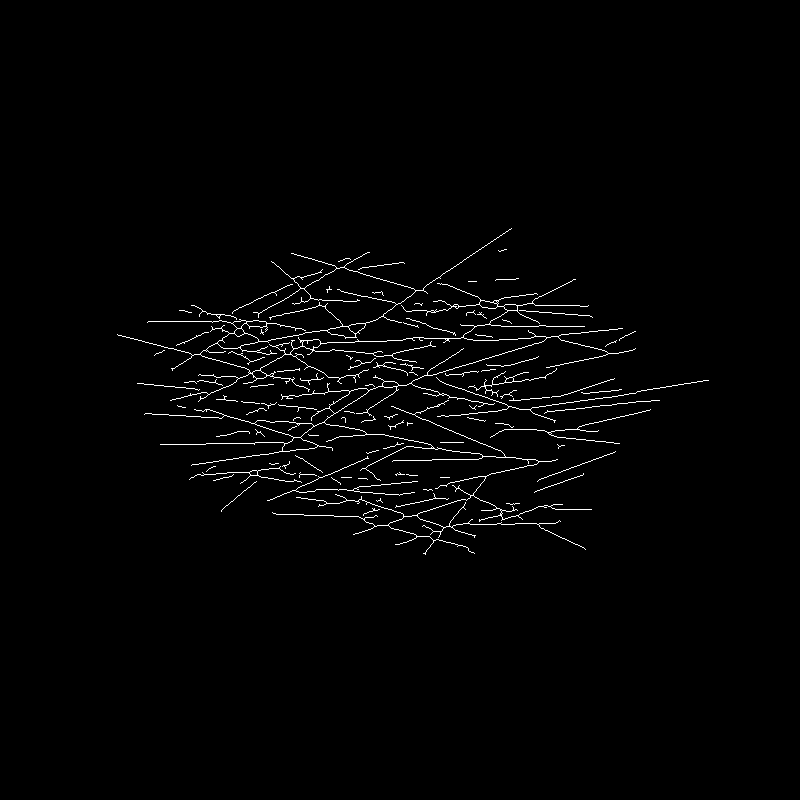

Supplement: S1 File — This comprises the FilamentSensor, the benchmark database, the output of the compared programs, several auxiliary scripts, and the evaluation results of the program outputs. (ZIP) [file pone.0126346.s001.zip › supporting_information/ground_truth/simulated/CID/simulated_cell_09_bin_img.png]

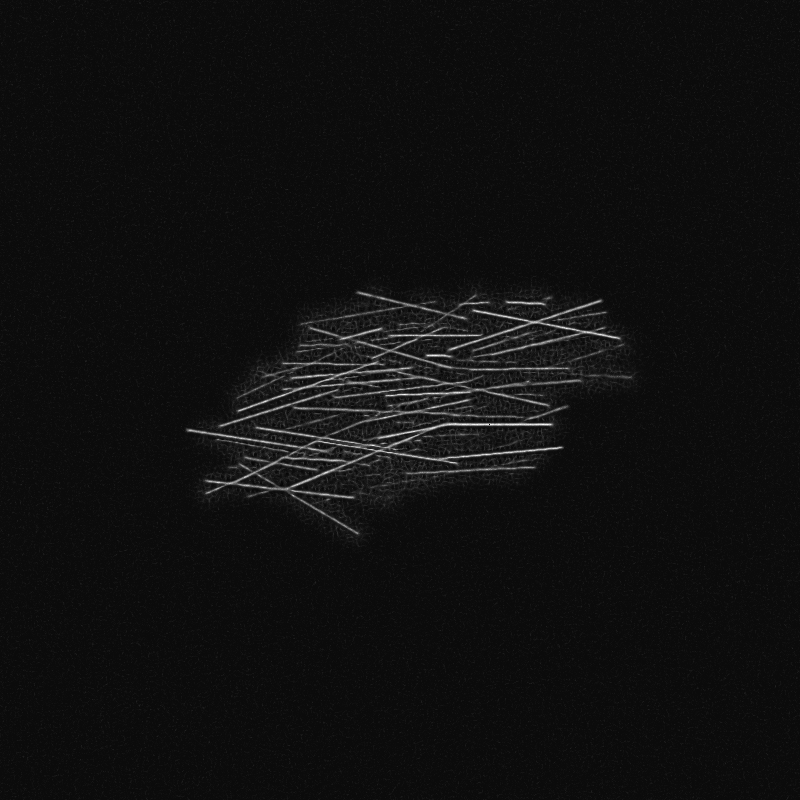

Supplement: S1 File — This comprises the FilamentSensor, the benchmark database, the output of the compared programs, several auxiliary scripts, and the evaluation results of the program outputs. (ZIP) [file pone.0126346.s001.zip › supporting_information/ground_truth/simulated/CID/simulated_cell_10_xcorr.png]

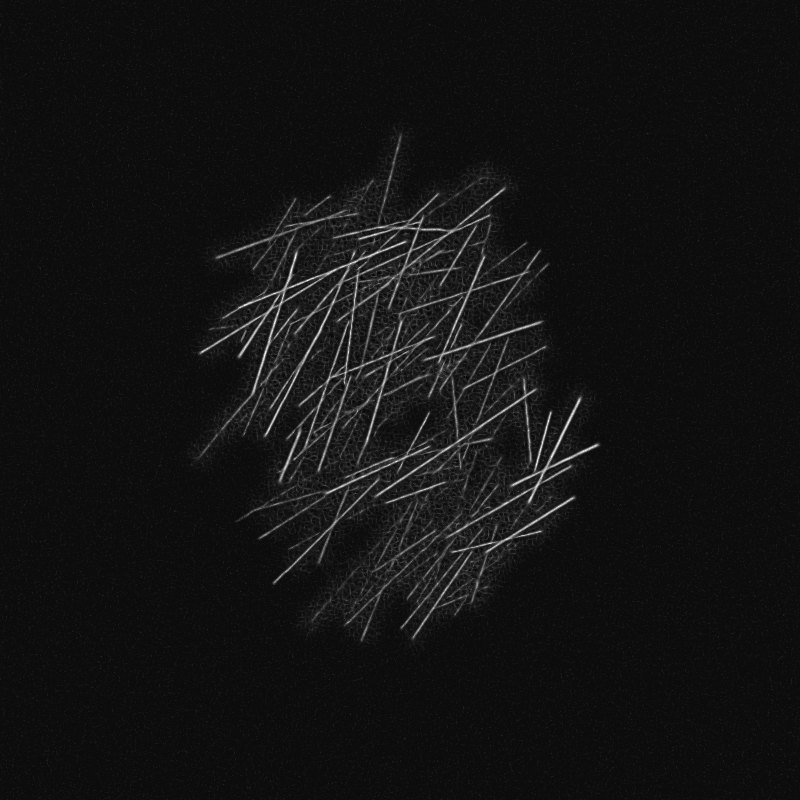

Supplement: S1 File — This comprises the FilamentSensor, the benchmark database, the output of the compared programs, several auxiliary scripts, and the evaluation results of the program outputs. (ZIP) [file pone.0126346.s001.zip › supporting_information/ground_truth/simulated/CID/simulated_cell_06_xcorr_scaled.png]

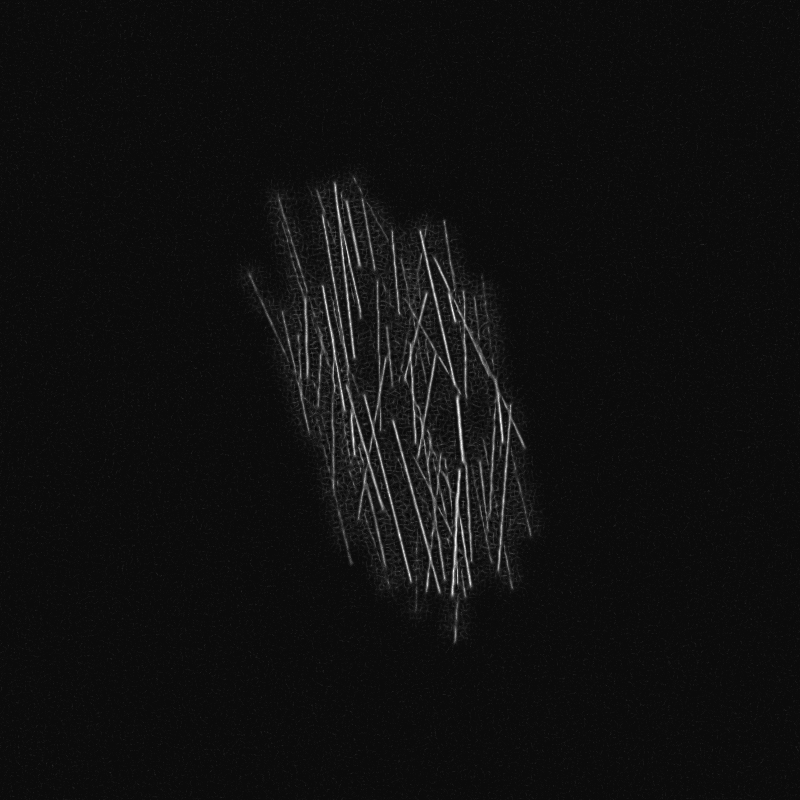

Supplement: S1 File — This comprises the FilamentSensor, the benchmark database, the output of the compared programs, several auxiliary scripts, and the evaluation results of the program outputs. (ZIP) [file pone.0126346.s001.zip › supporting_information/ground_truth/simulated/CID/simulated_cell_08_xcorr_scaled.png]

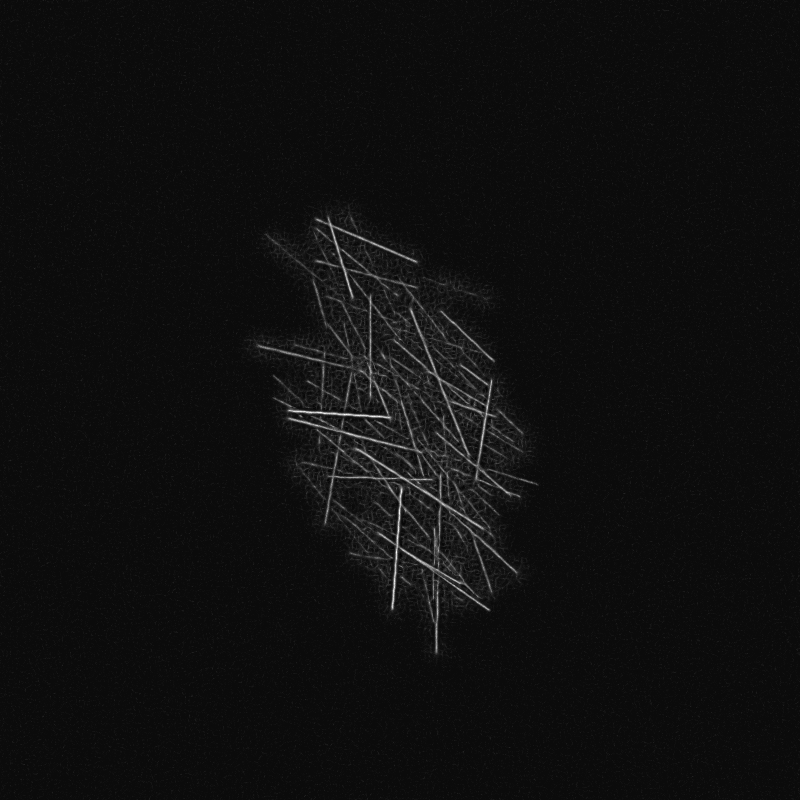

Supplement: S1 File — This comprises the FilamentSensor, the benchmark database, the output of the compared programs, several auxiliary scripts, and the evaluation results of the program outputs. (ZIP) [file pone.0126346.s001.zip › supporting_information/ground_truth/simulated/CID/simulated_cell_04_xcorr_scaled.png]

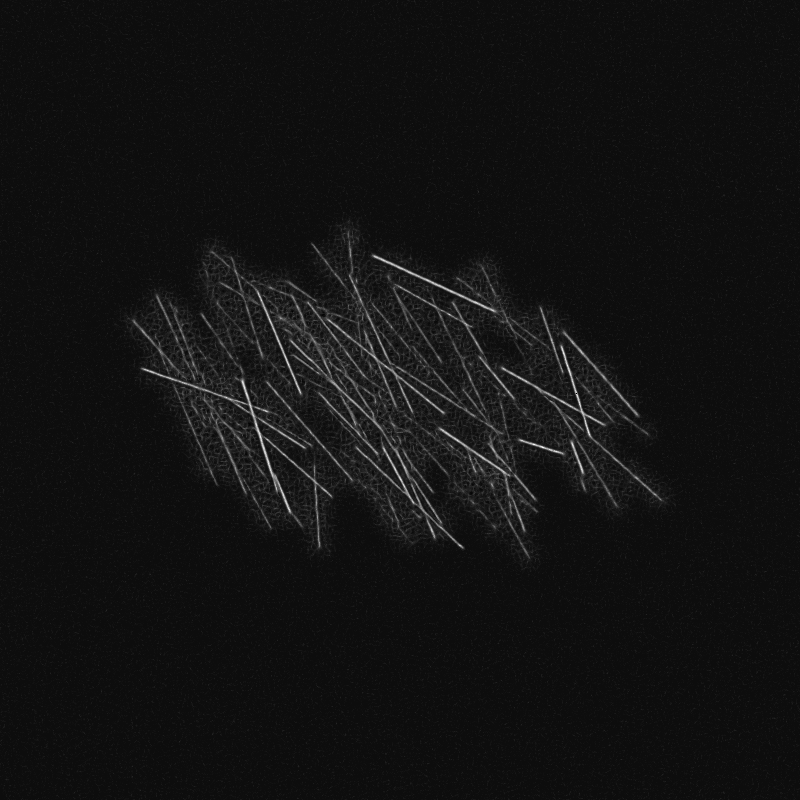

Supplement: S1 File — This comprises the FilamentSensor, the benchmark database, the output of the compared programs, several auxiliary scripts, and the evaluation results of the program outputs. (ZIP) [file pone.0126346.s001.zip › supporting_information/ground_truth/simulated/CID/simulated_cell_03_xcorr.png]

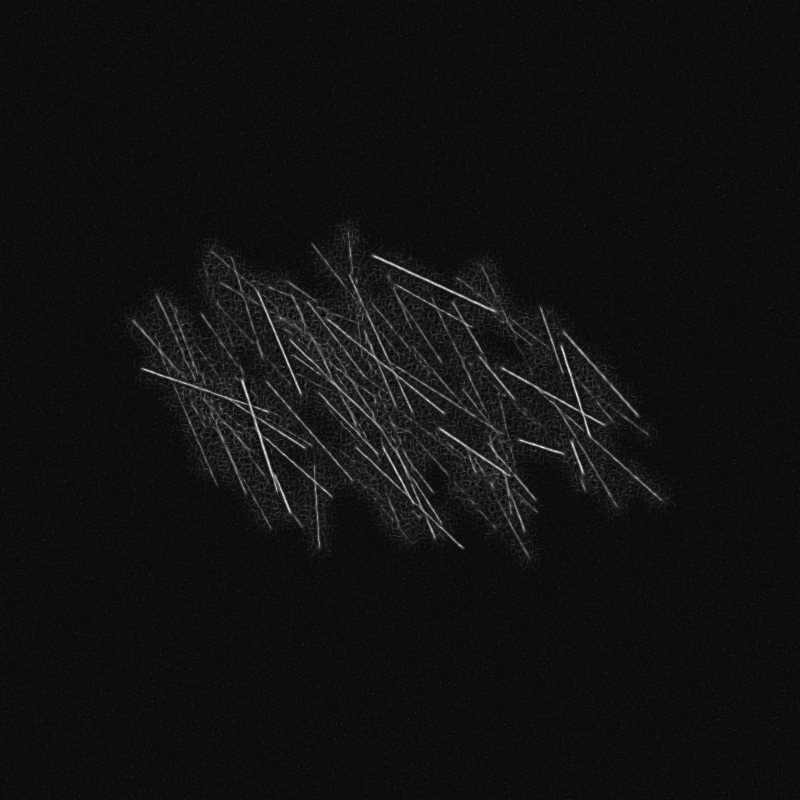

Supplement: S1 File — This comprises the FilamentSensor, the benchmark database, the output of the compared programs, several auxiliary scripts, and the evaluation results of the program outputs. (ZIP) [file pone.0126346.s001.zip › supporting_information/ground_truth/simulated/CID/simulated_cell_03_xcorr_scaled.png]

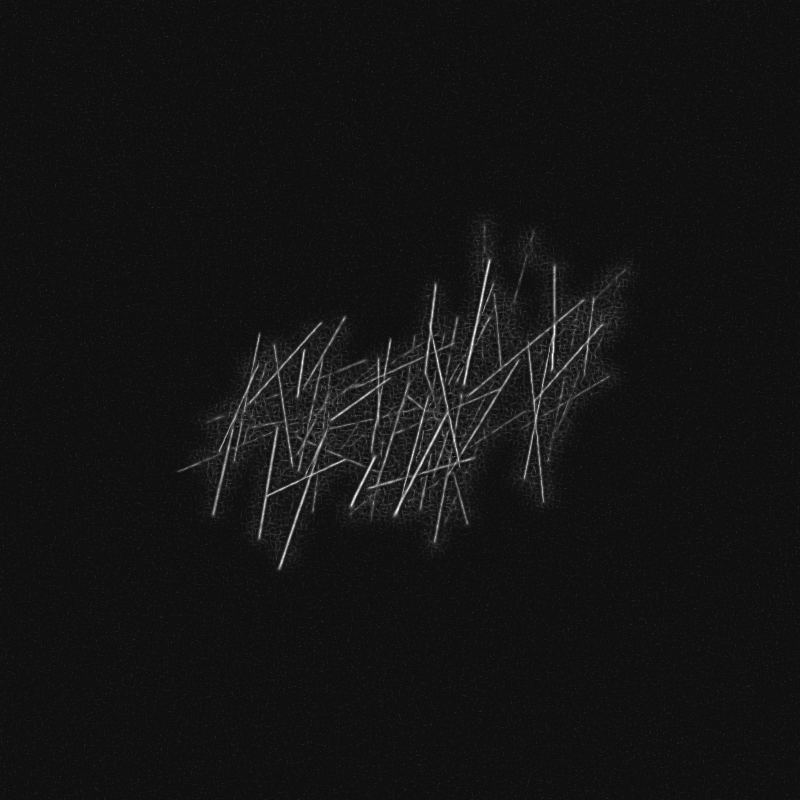

Supplement: S1 File — This comprises the FilamentSensor, the benchmark database, the output of the compared programs, several auxiliary scripts, and the evaluation results of the program outputs. (ZIP) [file pone.0126346.s001.zip › supporting_information/ground_truth/simulated/CID/simulated_cell_02_xcorr_scaled.png]

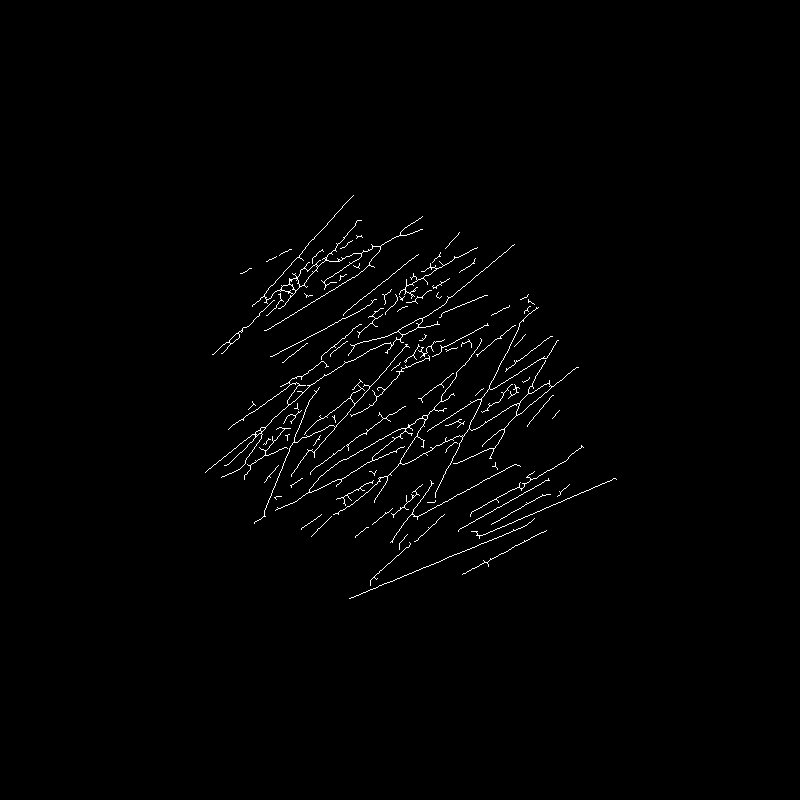

Supplement: S1 File — This comprises the FilamentSensor, the benchmark database, the output of the compared programs, several auxiliary scripts, and the evaluation results of the program outputs. (ZIP) [file pone.0126346.s001.zip › supporting_information/ground_truth/simulated/CID/simulated_cell_05_bin_img.png]

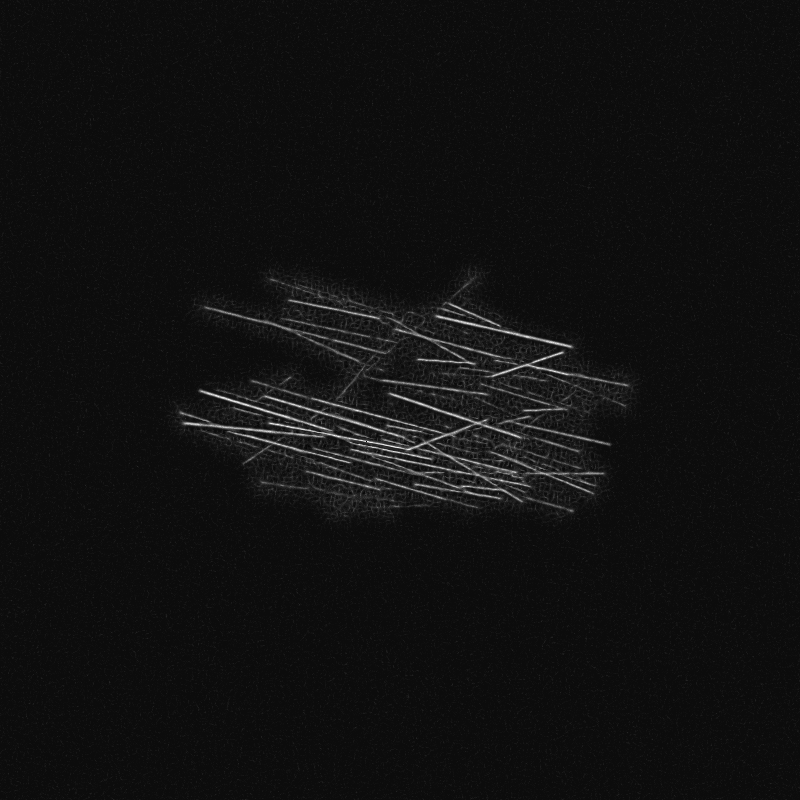

Supplement: S1 File — This comprises the FilamentSensor, the benchmark database, the output of the compared programs, several auxiliary scripts, and the evaluation results of the program outputs. (ZIP) [file pone.0126346.s001.zip › supporting_information/ground_truth/simulated/CID/simulated_cell_01_xcorr.png]

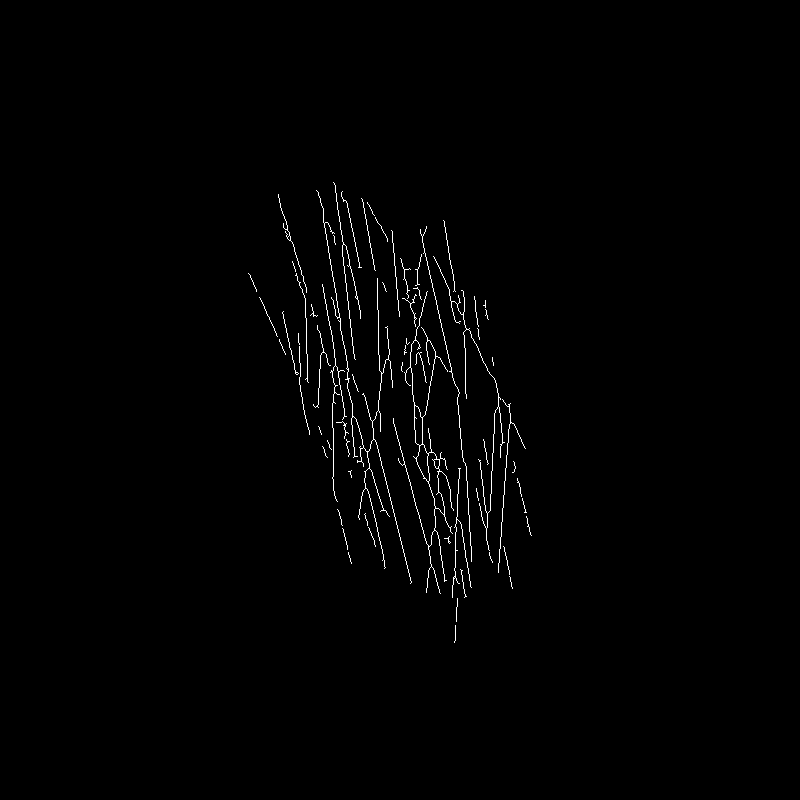

Supplement: S1 File — This comprises the FilamentSensor, the benchmark database, the output of the compared programs, several auxiliary scripts, and the evaluation results of the program outputs. (ZIP) [file pone.0126346.s001.zip › supporting_information/ground_truth/simulated/CID/simulated_cell_08_bin_img.png]

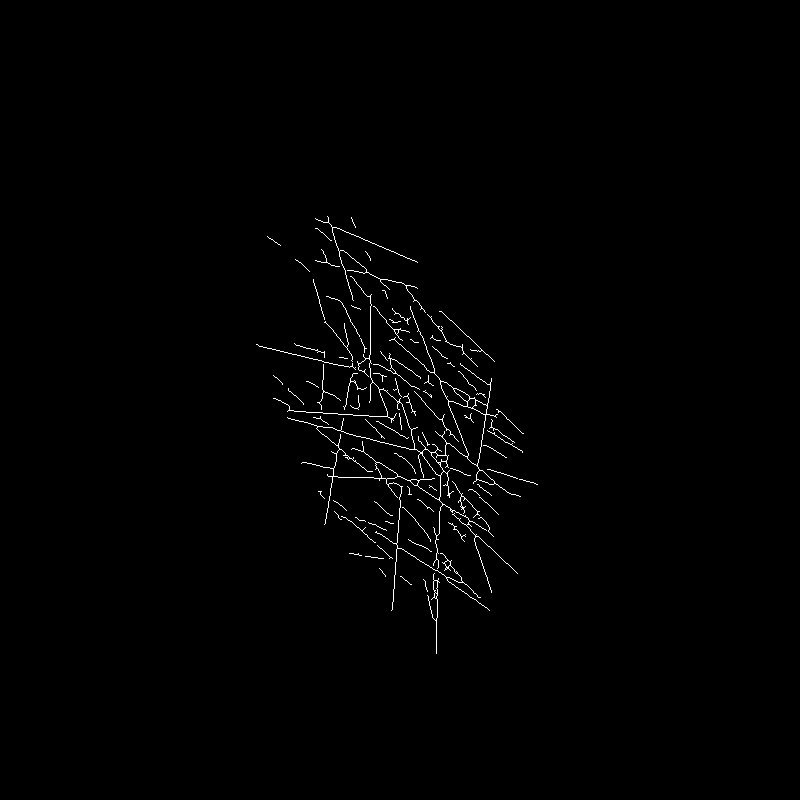

Supplement: S1 File — This comprises the FilamentSensor, the benchmark database, the output of the compared programs, several auxiliary scripts, and the evaluation results of the program outputs. (ZIP) [file pone.0126346.s001.zip › supporting_information/ground_truth/simulated/CID/simulated_cell_04_bin_img.png]

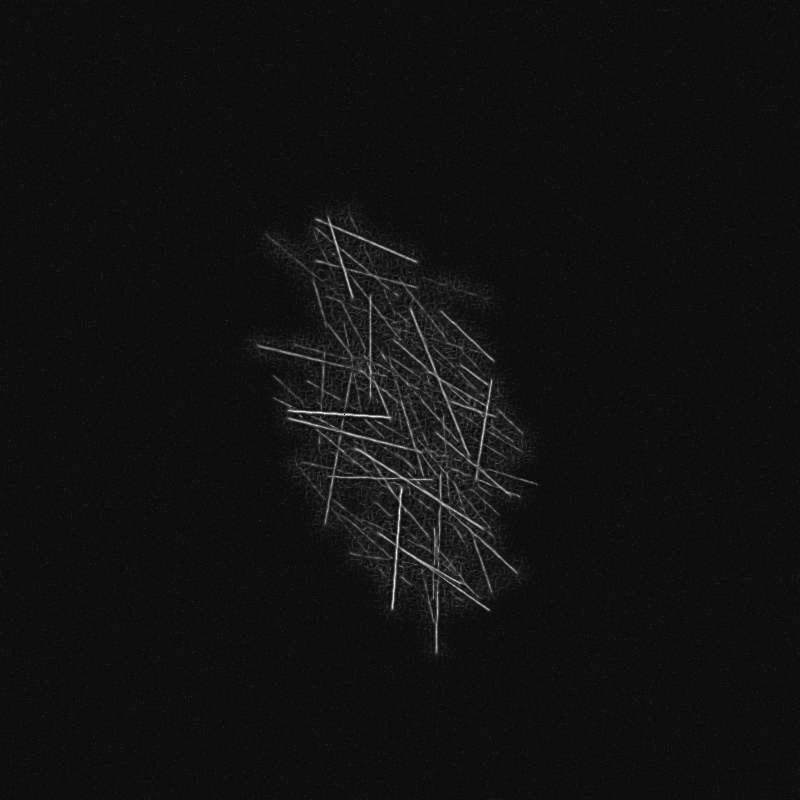

Supplement: S1 File — This comprises the FilamentSensor, the benchmark database, the output of the compared programs, several auxiliary scripts, and the evaluation results of the program outputs. (ZIP) [file pone.0126346.s001.zip › supporting_information/ground_truth/simulated/CID/simulated_cell_04_xcorr.png]

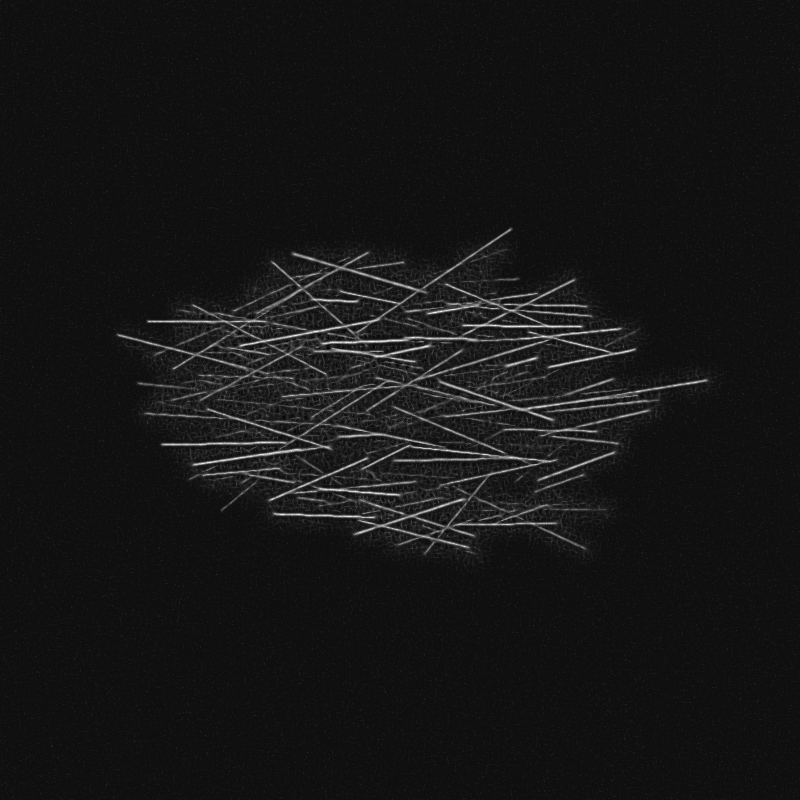

Supplement: S1 File — This comprises the FilamentSensor, the benchmark database, the output of the compared programs, several auxiliary scripts, and the evaluation results of the program outputs. (ZIP) [file pone.0126346.s001.zip › supporting_information/ground_truth/simulated/CID/simulated_cell_09_xcorr_scaled.png]

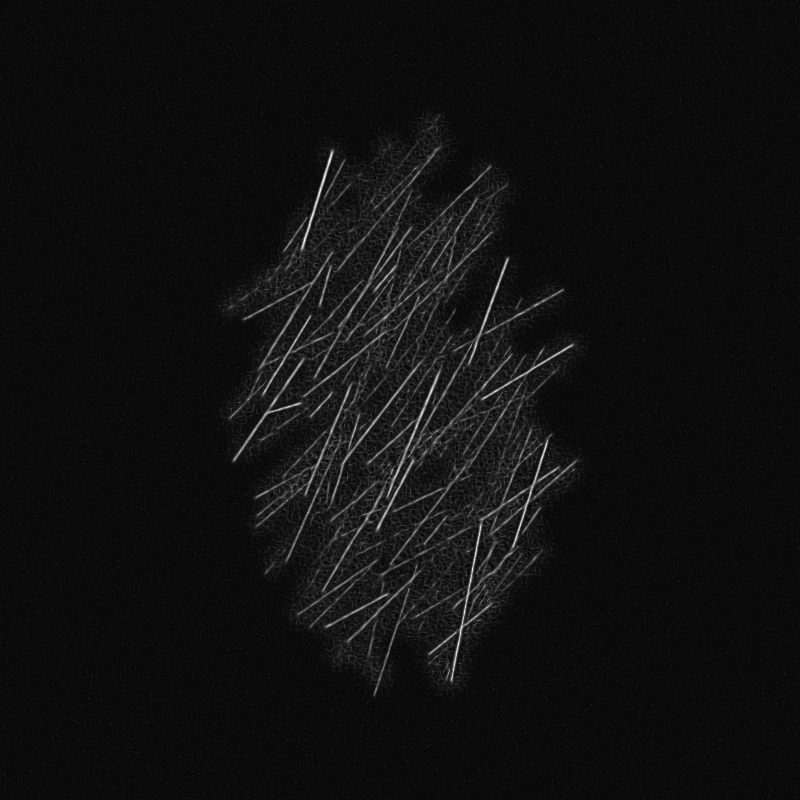

Supplement: S1 File — This comprises the FilamentSensor, the benchmark database, the output of the compared programs, several auxiliary scripts, and the evaluation results of the program outputs. (ZIP) [file pone.0126346.s001.zip › supporting_information/ground_truth/simulated/CID/simulated_cell_07_xcorr_scaled.png]

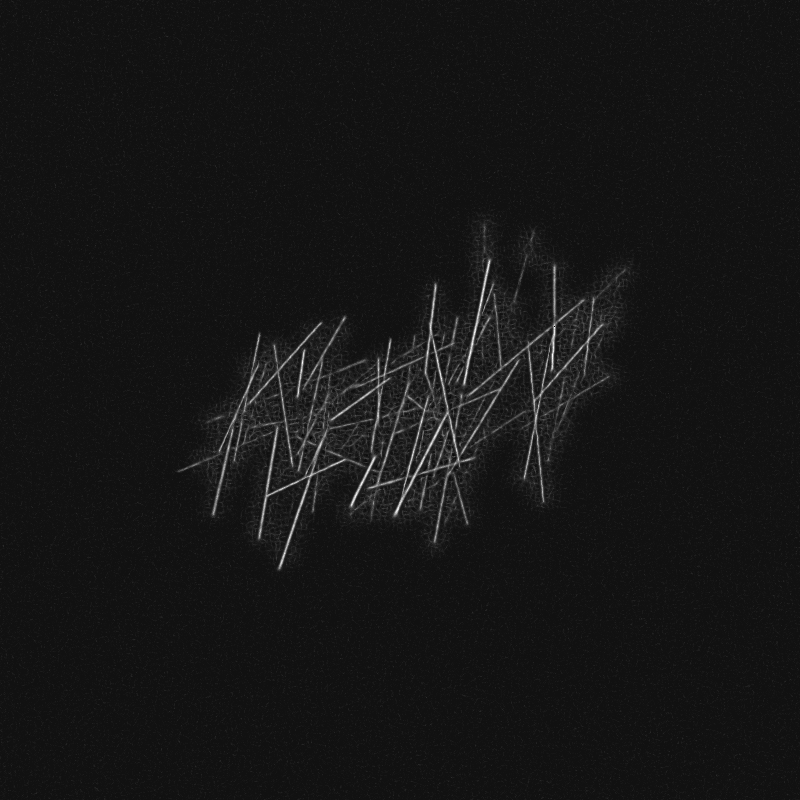

Supplement: S1 File — This comprises the FilamentSensor, the benchmark database, the output of the compared programs, several auxiliary scripts, and the evaluation results of the program outputs. (ZIP) [file pone.0126346.s001.zip › supporting_information/ground_truth/simulated/CID/simulated_cell_02_xcorr.png]

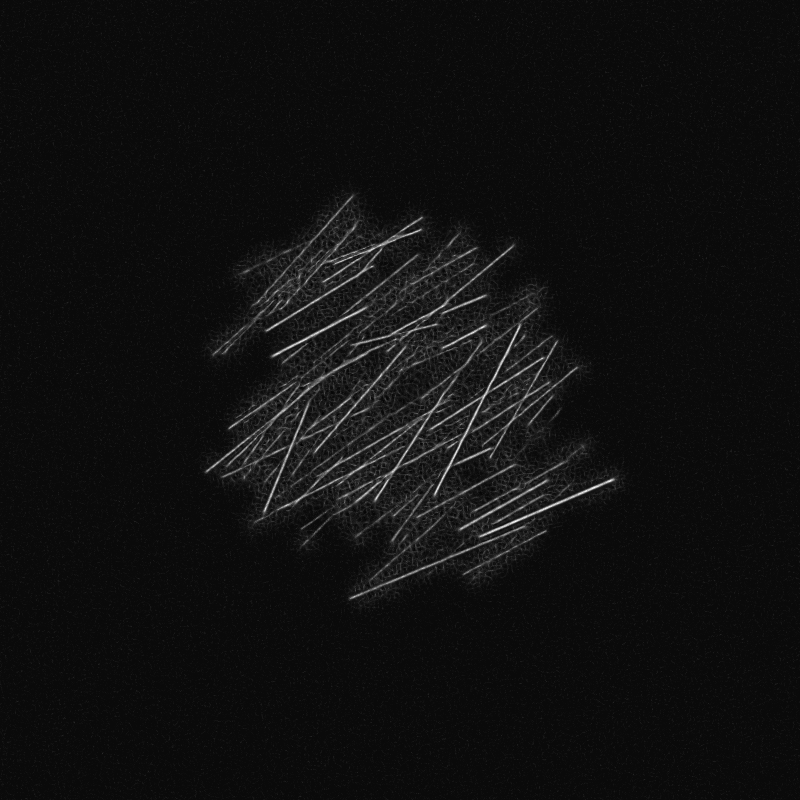

Supplement: S1 File — This comprises the FilamentSensor, the benchmark database, the output of the compared programs, several auxiliary scripts, and the evaluation results of the program outputs. (ZIP) [file pone.0126346.s001.zip › supporting_information/ground_truth/simulated/CID/simulated_cell_05_xcorr_scaled.png]

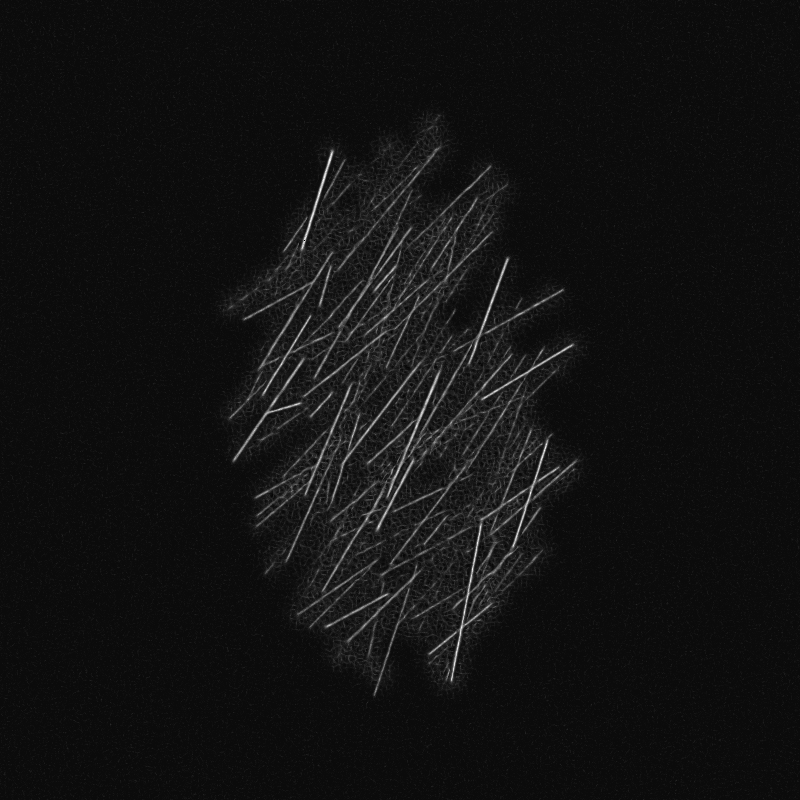

Supplement: S1 File — This comprises the FilamentSensor, the benchmark database, the output of the compared programs, several auxiliary scripts, and the evaluation results of the program outputs. (ZIP) [file pone.0126346.s001.zip › supporting_information/ground_truth/simulated/CID/simulated_cell_07_xcorr.png]

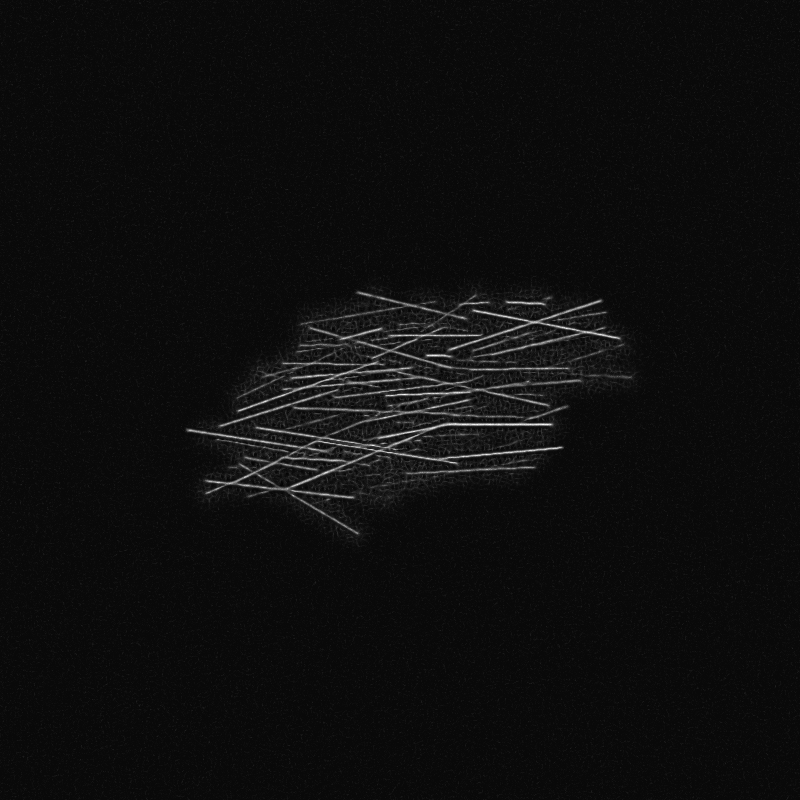

Supplement: S1 File — This comprises the FilamentSensor, the benchmark database, the output of the compared programs, several auxiliary scripts, and the evaluation results of the program outputs. (ZIP) [file pone.0126346.s001.zip › supporting_information/ground_truth/simulated/CID/simulated_cell_10_xcorr_scaled.png]

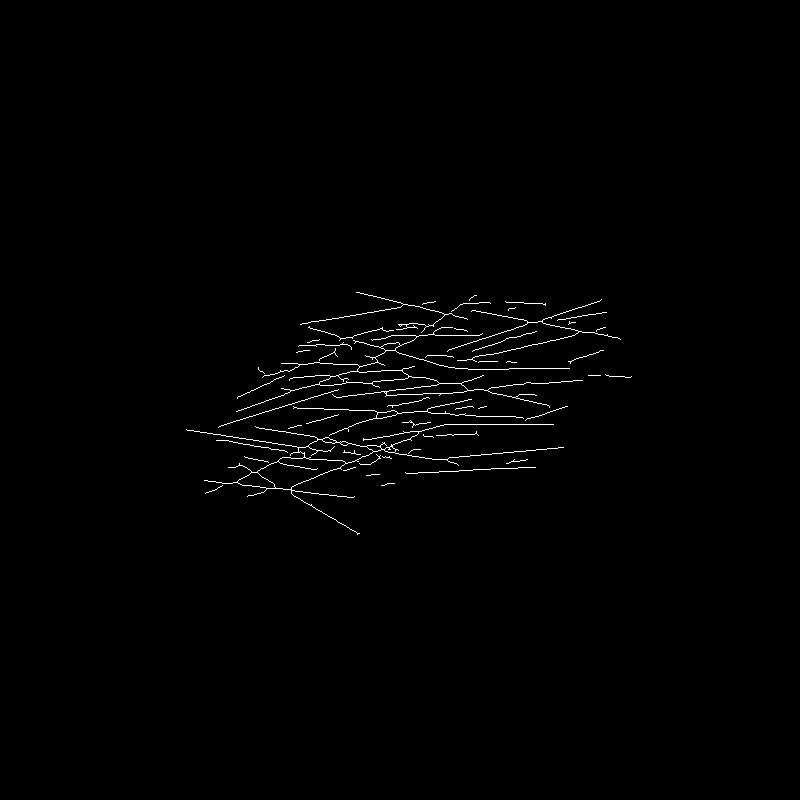

Supplement: S1 File — This comprises the FilamentSensor, the benchmark database, the output of the compared programs, several auxiliary scripts, and the evaluation results of the program outputs. (ZIP) [file pone.0126346.s001.zip › supporting_information/ground_truth/simulated/CID/simulated_cell_10_bin_img.png]

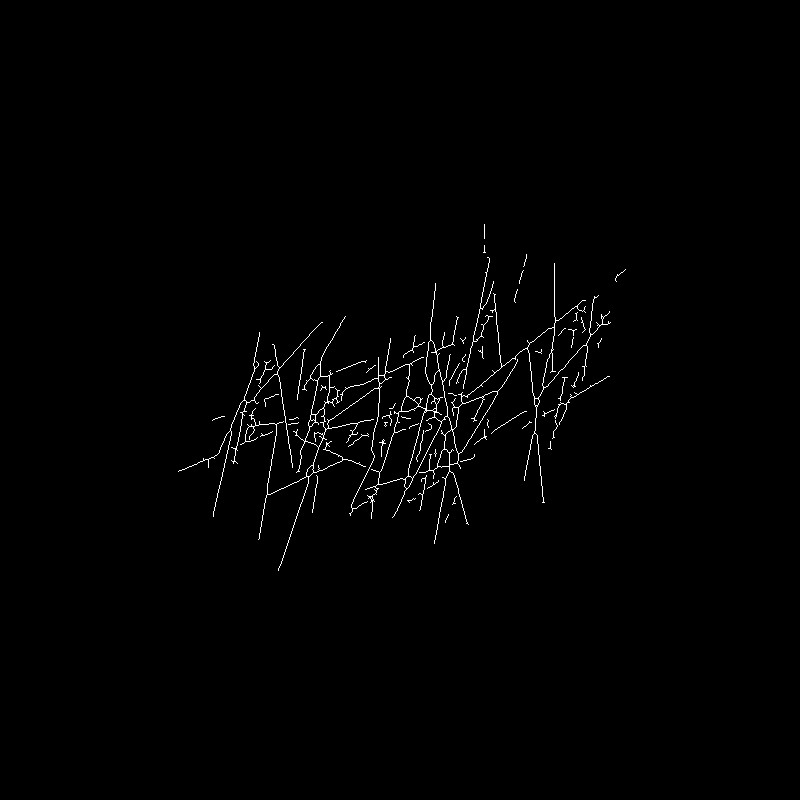

Supplement: S1 File — This comprises the FilamentSensor, the benchmark database, the output of the compared programs, several auxiliary scripts, and the evaluation results of the program outputs. (ZIP) [file pone.0126346.s001.zip › supporting_information/ground_truth/simulated/CID/simulated_cell_02_bin_img.png]

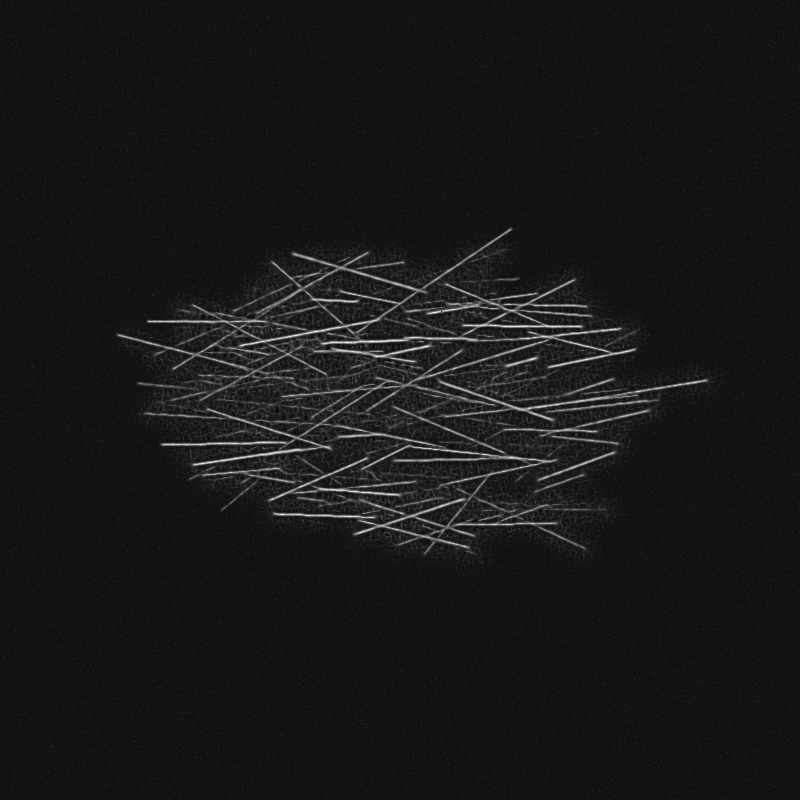

Supplement: S1 File — This comprises the FilamentSensor, the benchmark database, the output of the compared programs, several auxiliary scripts, and the evaluation results of the program outputs. (ZIP) [file pone.0126346.s001.zip › supporting_information/ground_truth/simulated/CID/simulated_cell_09_xcorr.png]

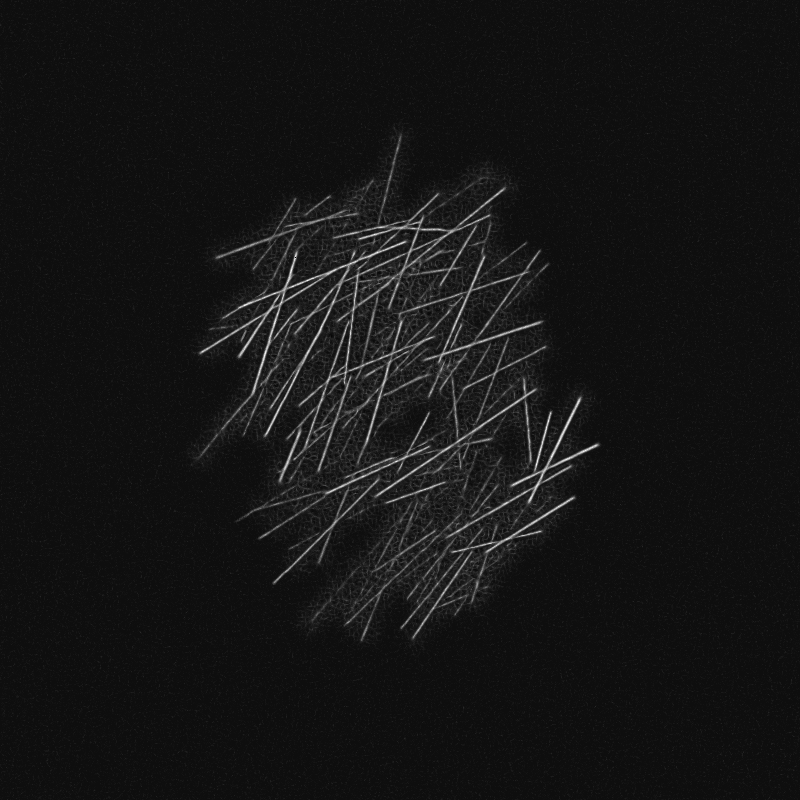

Supplement: S1 File — This comprises the FilamentSensor, the benchmark database, the output of the compared programs, several auxiliary scripts, and the evaluation results of the program outputs. (ZIP) [file pone.0126346.s001.zip › supporting_information/ground_truth/simulated/CID/simulated_cell_06_xcorr.png]

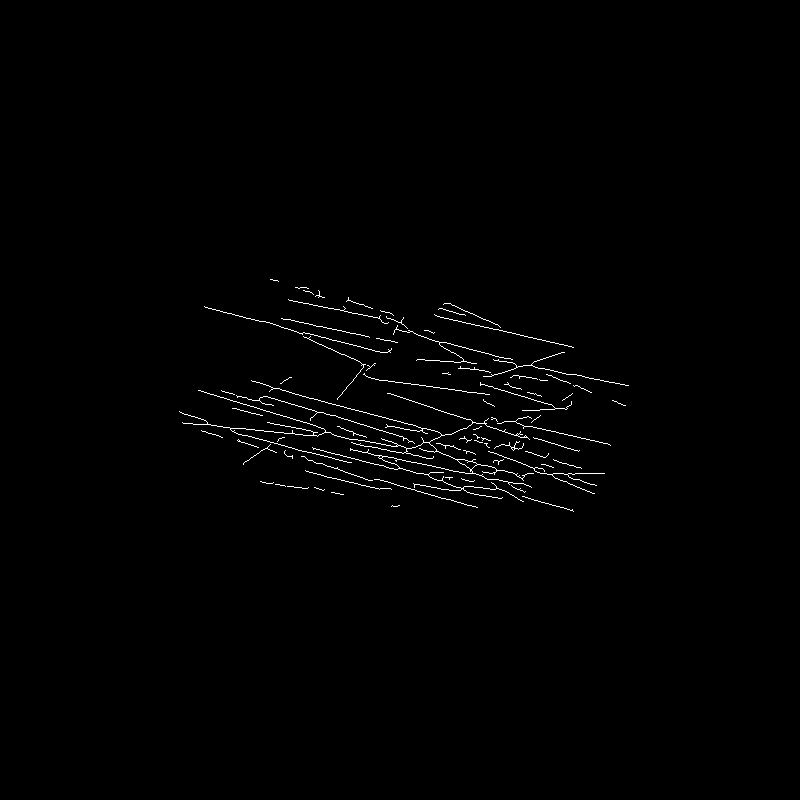

Supplement: S1 File — This comprises the FilamentSensor, the benchmark database, the output of the compared programs, several auxiliary scripts, and the evaluation results of the program outputs. (ZIP) [file pone.0126346.s001.zip › supporting_information/ground_truth/simulated/CID/simulated_cell_01_bin_img.png]

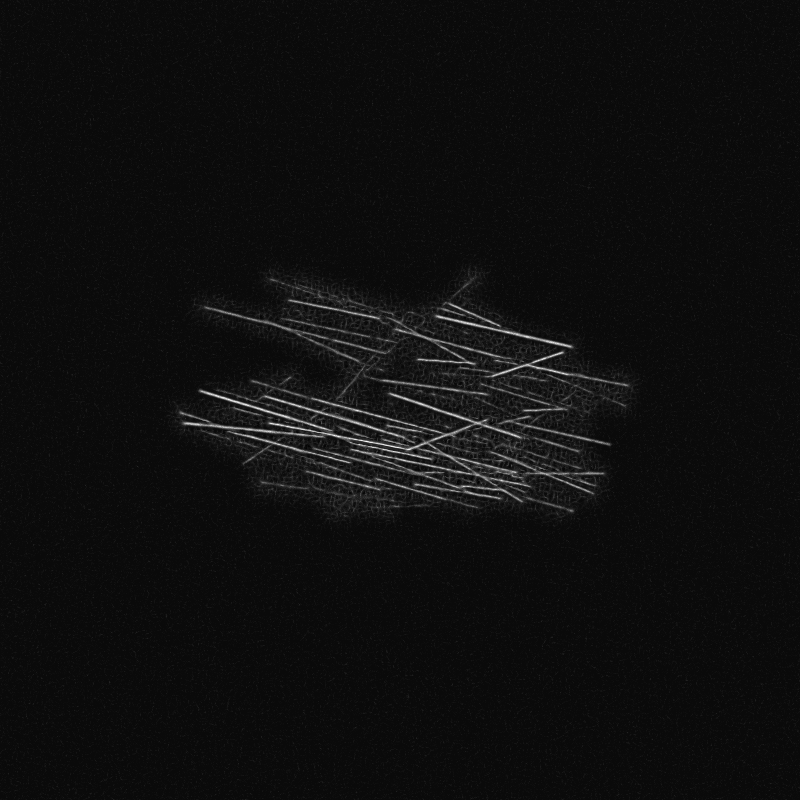

Supplement: S1 File — This comprises the FilamentSensor, the benchmark database, the output of the compared programs, several auxiliary scripts, and the evaluation results of the program outputs. (ZIP) [file pone.0126346.s001.zip › supporting_information/ground_truth/simulated/CID/simulated_cell_01_xcorr_scaled.png]

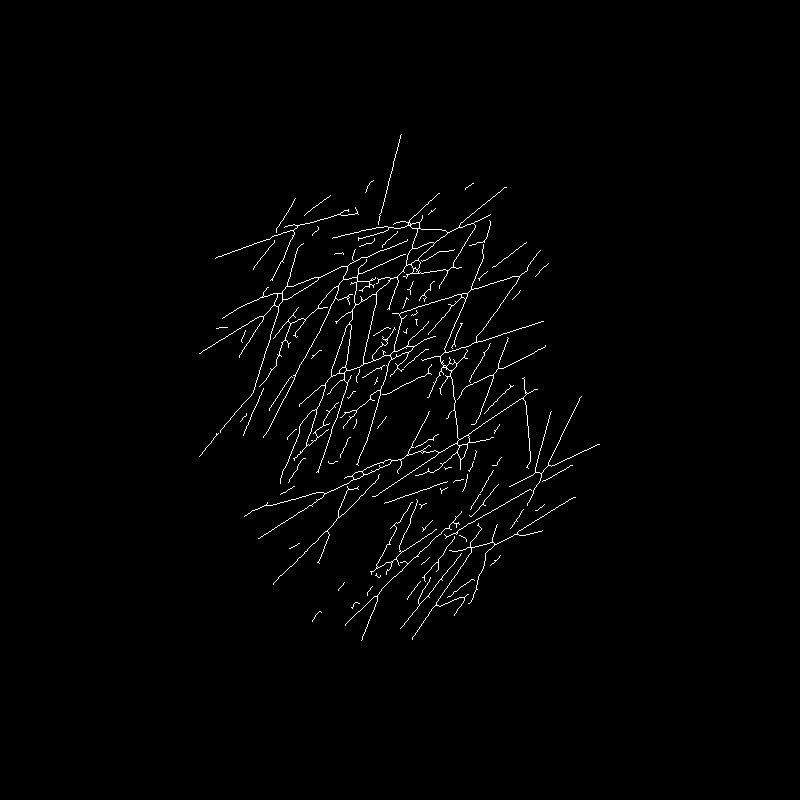

Supplement: S1 File — This comprises the FilamentSensor, the benchmark database, the output of the compared programs, several auxiliary scripts, and the evaluation results of the program outputs. (ZIP) [file pone.0126346.s001.zip › supporting_information/ground_truth/simulated/CID/simulated_cell_06_bin_img.png]
